# ARVib suppresses growth of advanced prostate cancer via inhibition of androgen receptor signaling

**DOI:** 10.1038/s41388-021-01914-2

**Published:** 2021-07-16

**Authors:** Chengfei Liu, Cameron M. Armstrong, Shu Ning, Joy C. Yang, Wei Lou, Alan P. Lombard, Jinge Zhao, Chun-Yi Wu, Aiming Yu, Christopher P. Evans, Clifford G. Tepper, Pui-kai Li, Allen C. Gao

**Affiliations:** 1 Department of Urologic Surgery, University of California Davis, Davis, CA USA; 2 UC Davis Comprehensive Cancer Center, University of California Davis, Davis, CA USA; 3 Department of Biochemistry and Molecular Medicine, University of California Davis, Davis, CA USA; 4 Division of Medicinal Chemistry and Pharmacognosy, College of Pharmacy, The Ohio State University, Columbus, OH USA; 5 VA Northern California Health Care System, Sacramento, CA USA

**Keywords:** Prostate cancer, Phenotypic screening

## Abstract

Targeting androgen signaling with the second-generation anti-androgen drugs, such as enzalutamide (Enza), abiraterone (Abi), apalutamide (Apal), and darolutamide (Daro), is the mainstay for the treatment of castration-resistant prostate cancer (CRPC). While these treatments are effective initially, resistance occurs frequently. Continued expression of androgen receptor (AR) and its variants such as AR-V7 despite AR-targeted therapy contributes to treatment resistance and cancer progression in advanced CRPC patients. This highlights the need for new strategies blocking continued AR signaling. Here, we identify a novel AR/AR-V7 degrader (ARVib) and found that ARVib effectively degrades AR/AR-V7 protein and attenuates AR/AR-V7 downstream target gene expression in prostate cancer cells. Mechanistically, ARVib degrades AR/AR-V7 protein through the ubiquitin-proteasome pathway mediated by HSP70/STUB1 machinery modulation. ARVib suppresses HSP70 expression and promotes STUB1 nuclear translocation, where STUB1 binds to AR/AR-V7 and promotes its ubiquitination and degradation. ARVib significantly inhibits resistant prostate tumor growth and improves enzalutamide treatment in vitro and in vivo. These data suggest that ARVib has potential for development as an AR/AR-V7 degrader to treat resistant CRPC.

## Introduction

Targeting androgen signaling via androgen deprivation therapy (ADT) is the first line treatment for prostate cancer (PCa). While initially effective, the majority of men eventually develop castration-resistant prostate cancer (CRPC). Enzalutamide (Enza), abiraterone (Abi), apalutamide (Apal), and darolutamide (Daro) are the second-generation anti-androgen drugs used for the treatment of CRPC and function by inhibiting androgen receptor (AR) signaling [1–4]. Even though Enza and Abi have been shown to be effective initially, resistance to Enza and Abi occurs frequently. Considerable evidence from both clinical and experimental studies demonstrates that expression of AR-variant 7 (AR-V7) plays a vital role in promoting CRPC progression and induction of resistance to Enza and Abi therapy [5–7]. Furthermore, it has been shown that AR-V7 expression in cancer patients treated with Enza or Abi correlates to a significantly lower PSA response, shorter progression-free time, and lower overall survival time compared to patients who do not express AR-V7 [8, 9]. AR-V7 is a ligand-independent and constitutively activated transcriptional factor and is not targeted by either Enza or Abi [5, 10, 11]. Therefore, there is an urgent need to develop novel agents and strategies to block AR/AR-V7 to overcome resistance.

Disruption of protein homeostasis (proteostasis) in cells causes aberrant folding and aggregation of pro-oncogenic proteins which is integrated by molecular chaperones, the ubiquitin-proteasome system, and their regulators [12, 13]. Cancer cells are able to escape from apoptosis, veering towards tumor growth, metastasis, and drug resistance by exploiting proteostasis perturbations to activate oncogenic pathways [14]. Despite great efforts being put in to discovering small molecules which block biological function of oncogenic drivers, inducing oncogenic protein degradation through the ubiquitin-proteasome system has been emerging as an attractive strategy for cancer therapy [14].

Many studies have investigated ways to target persistent AR signaling with the goal to overcome resistance to AR-targeted therapies. These include finding methods to promote AR degradation, targeting the N-terminal domain and DNA binding domain of the AR, inhibition of AR synthesis and targeting AR co-regulators [15–19]. Our previous study reported that niclosamide, an FDA-approved antihelminthic drug, functions as a potent inhibitor of AR/AR-V7 via protein degradation by activation of the ubiquitination-proteolytic pathway [20, 21]. However, niclosamide has poor water solubility and bioavailability which limit it from systemic administration for patients.

In the present study, we synthesized a library of niclosamide analogs according to predicted bioavailability in order to improve its bioavailability and potency. We have identified several analogs (named ARVib) that can target AR/AR-V7 for degradation. In particular, analog #7 (ARVib-7) has better bioavailability than niclosamide and can inhibit prostate cancer cell proliferation and AR transcriptional activity. We demonstrate that ARVib degrades AR/AR-V7 protein expression through the ubiquitin-proteasome pathway mediated by HSP70/STUB1 machinery modulation. ARVib suppresses HSP70 expression and promotes STUB1 nuclear translocation, where STUB1 binds to AR/AR-V7 and promotes its ubiquitination and degradation. ARVib significantly inhibited resistant prostate tumor growth and improved Enza treatment in vitro and in vivo.

## Results

### Synthesis of niclosamide analogs and identification of potent inhibitors of AR/AR variants

**Fig. 1:**
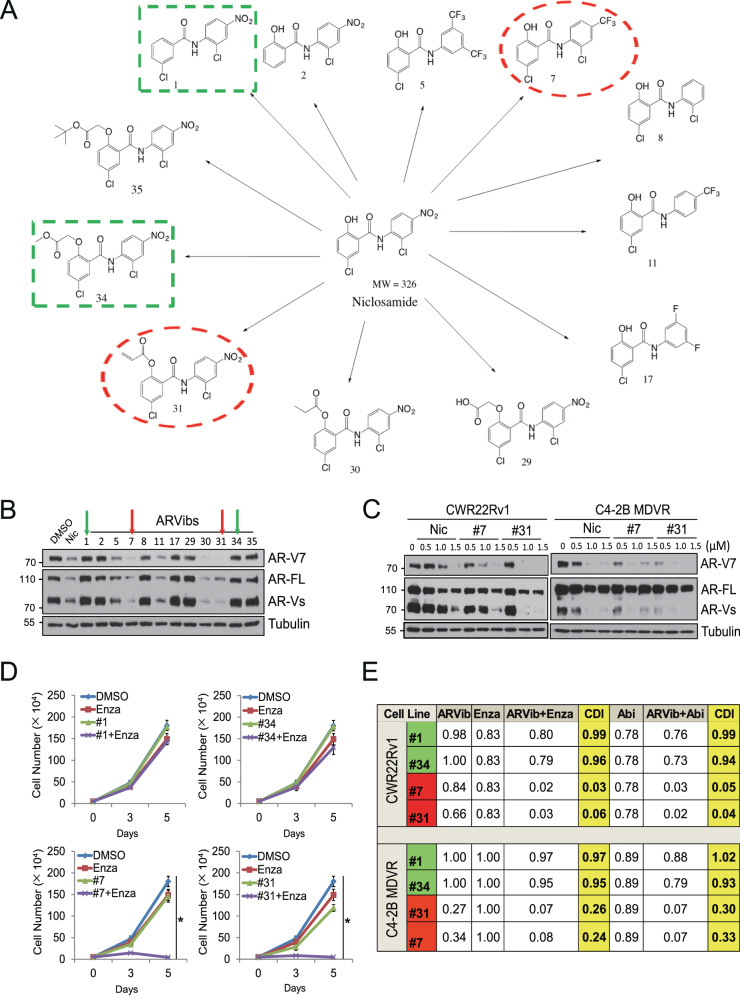
Synthesis of niclosamide analogs and identification of potent inhibitors of AR/AR variants. **A** The chemical structure of niclosamide and newly synthesized ARVibs. The red box indicates the structures of #7 and #31, the green box indicates chemical structures of #1 and #34. **B** CWR22Rv1 cells were treated with 1 µM ARVib for 16 h and whole-cell lysates were collected and subjected to western blot. **C** C4-2B MDVR and CWR22Rv1 cells were treated with different doses of ARVibs for 16 h and whole-cell lysates were collected and subjected to western blot. **D** CWR22Rv1 cells were treated with enzalutamide with or without ARVibs (#1, #34, #7, and #31), total cell numbers were determined at 0, 3, 5 days. **E** The CDI of enzalutamide or abiraterone with ARVibs in CWR22Rv1 and C4-2B MDVR cells were calculated. *p < 0.05. Results are the mean of three independent experiments (±S.D.). AR-FL: full-length AR, AR-Vs AR-Variants, Enza enzalutamide, Abi abiraterone, Nic niclosamide, CDI: coefficient of drug interaction.

Our previous studies discovered that niclosamide is a potent inhibitor of AR/AR variants and functions to enhance Enza and Abi treatment [20, 21]. However, one of the limitations of niclosamide is that it has poor bioavailability, which may limit it from systemic penetration as an anticancer agent in humans. To improve the bioavailability and potency of niclosamide, we synthesized a library of its analogs according to predicted bioavailability. The chemical structures of the representative newly synthesized compounds are illustrated in Fig. 1A. Since niclosamide can inhibit AR/AR-V7 expression, we examined the effects of these newly synthesized analogs on AR and AR-variants expression. CWR22Rv1 prostate cancer cells that express both AR and AR variants were treated with the newly synthezied compounds as indicated (1 μM for 16 h). Protein lysates were analyzed for AR and AR-variant expression. Figure1B shows that compounds −5, −7, −11, −30, and −31 inhibited AR and AR-V7 expression. Among them, niclosamide inhibited AR by 89.73% and AR-V7 by 85.85%, #7 inhibited AR by 91.64% and AR-V7 by 92.06%, #31 inhibited AR by 86.49% and AR-V7 by 99.78%. In contrast, compounds #1, 2, 8, 17, 29, 34, 35 had little or no effect on the expression of the AR and AR variants. Further studies demonstrate that compounds −7 and −31 inhibited AR and AR-V7 expression in a dose-dependent manner in CWR22Rv1 cells. As shown in Fig. 1C left, 1 µM niclosamide inhibited AR by 84.42% and AR-V7 by 80.68% in CWR22Rv1 cells, #7 inhibited AR by 93.59% and AR-V7 by 89.20%, #31 inhibited AR by 85.12% and AR-V7 by 99.22%. Similar results were also observed in C4-2B MDVR cells (Fig. 1C right). Thus, we chose compound #7 and #31 for further characterization as potent inhibitors of AR/AR variants and named them ARVib (AR/AR-variant inhibitor).

Since AR-V7 is critically involved in driving resistance to anti-androgens such as enzalutamide and abiraterone, we hypothesized that compounds with the ability to inhibit AR-V7 would synergize with anti-androgens and improve their therapeutic activity in resistant prostate cancer. To test this hypothesis, we determined the effects of these compounds at sub low dose (0.25 μM) either alone or in combination with Enza or Abi on cell growth. The effects of ARVib-7 and ARVib-31 (which inhibit AR-V7 expression) on cell growth of resistant prostate cancer cells were compared to the effects of compounds #1, and #34 (which do not inhibit AR-V7 expression). As shown in Fig .1D and Supplementary Fig. 1A, CWR22Rv1 cells are resistant to both Enza and Abi. Compounds #1 and #34 alone were not able to inhibit growth of CWR22Rv1 cells. The combination of either compounds #1 or #34 with Enza or Abi also did not inhibit the growth of CWR22Rv1 cells. In contrast, combination of either ARVib-7 or ARVib-31 with Enza or Abi significantly inhibited the growth of CWR22Rv1 cells. Similar results were observed in Enza-resistant C4-2B MDVR cells, in which ARVib-7 and ARVib-31, but not compounds #1 and #34, were able to synergize with Enza/Abi (Supplementary Fig. 1B). Single treatments of ARVib-7 and ARVib-31 significantly suppressed the cell growth in 5 days, and in combination with Enza/Abi, they further reduced the cell growth. The coefficient of drug interaction (CDI) of ARVib-7, ARVib-31 in combination with Enza or Abi treatment were below 1.0, suggesting drug synergism (Fig. 1E). We also performed qPCR on mRNA isolated from MDVR cells treated with #7 in combination with Enza/Abi for 5 days to determine the effects on AR/AR-V7 target gene expression. As shown in Supplementary Fig. 1C, single treatment of #7 decreased the AR/AR-V7 targeted gene expression and combination of Enza/Abi with #7 further suppressed gene expression. Collectively, we demonstrated that compounds that have the ability to inhibit AR-V7 expression (ARVib-7, ARVib-31) can synergize with either Enza or Abi, while compounds that fail to inhibit AR-V7 expression (#1, #34) are not able to synergize with either Enza or Abi.

### ARVib disrupts AR/AR-V7 gene program in Enza-resistant prostate cancer cells

**Fig. 2:**
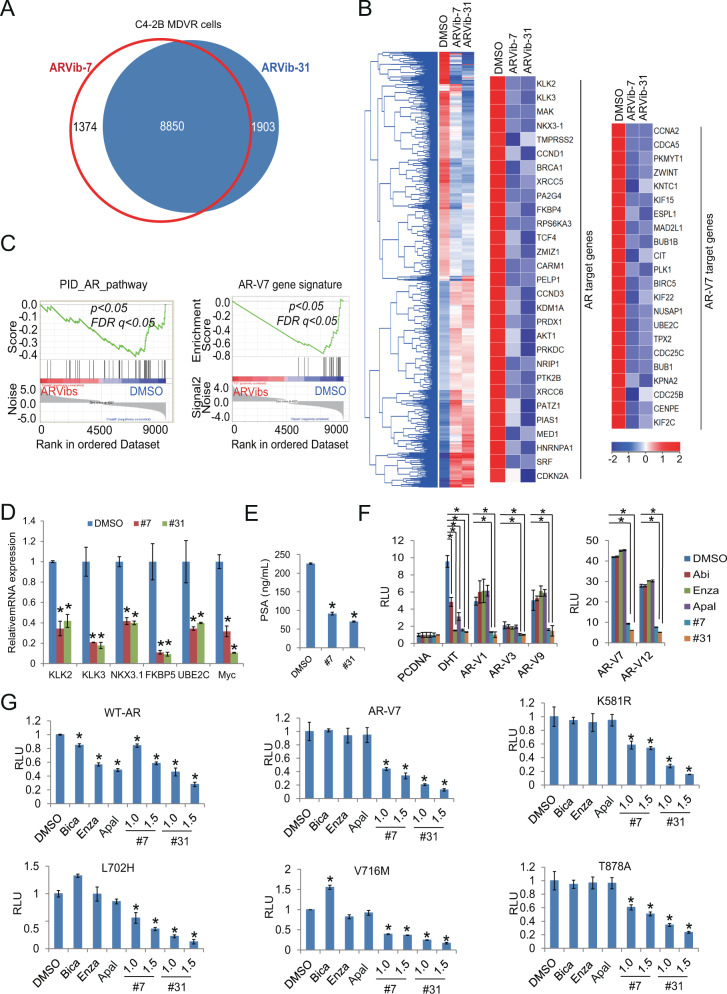
ARVib disrupts the AR/AR-V7 gene expression program in enzalutamide-resistant prostate cancer cells. **A** Venn diagram of RNA-seq results depicting the overlap of differentially expressed genes (DEGs) in C4-2B MDVR cells treated with 1.5 µM ARVib-7 (vs. DMSO) or 1.5 µM ARVib-31 (vs. DMSO) for 24 h. **B** Hierarchical clustering and heatmap visualization of the differentially expressed genes (DEGs) in ARVib-treated C4-2B MDVR cells with fold change (FC) > 1.2, as compared to vehicle (DMSO). The genes are displayed in rows and the normalized expression counts per sample are displayed in columns. Red and blue coloring indicates upregulated and downregulated relative expression levels, respectively. Middle and right, AR-FL and AR-V7 activity-signature genes that were altered in expression are displayed [5]. **C** GSEA of C4**-**2B MDVR cells treated with ARVibs (relative to DMSO control) demonstrates enrichment for suppressed expression of genes comprising PID-AR pathway (left) and the AR-V7 (right) gene signatures. The latter signature was defined by genes that are preferentially upregulated by AR-V7 [7]. **D** qRT-PCR analysis of the indicated genes in C4-2B MDVR cells treated with DMSO or 1 µM ARVibs for 48 h. **E** C4-2B MDVR cells were treated with DMSO or 1 µM ARVibs in FBS conditions for 48 h, PSA level was determined by PSA ELISA. **F** C4-2B cells were transiently transfected with pcDNA with or without 1 nM DHT, AR-V1, AR-V3, AR-V7, AR-V9, and AR-V12 with PSA E/P-luciferase plasmids in CS-FBS conditions, and then treated with DMSO, 5 µM abiraterone, 20 µM enzalutamide, 20 µM apalutamide, or 1 µM ARVibs for 16 h. PSA luciferase activity was examined. **G** 293 cells were transiently transfected with vector, AR-V7, mutant AR plus PSA promoter luciferase plasmids in FBS condition, and then treated with anti-androgens or ARVibs for 16 h. PSA luciferase activity was determined. *p < 0.05. Results are the mean of three independent experiments (±S.D.). Apal apalutamide, Bica bicalutamide.

To explore the gene regulating mechanisms underlying ARVib treatment in drug resistant prostate cancer cells, we performed RNA-sequencing analyses using C4-2B MDVR cells treated with ARVib-7 and ARVib-31 to identify gene programs affected by treatment. There are 10,224 genes and 10,753 genes that were differentially expressed in ARVib-7 and ARVib-31 treated C4-2B MDVR cells, respectively, and 8850 genes that were commonly regulated by both ARVib-7 and ARVib-31 (Fold change > 1.2; Fig. 2A). The top pathways upregulated by these compounds include unfolded protein response (UPR), the p53 pathway, hypoxia, and the apoptosis pathway. The downregulated pathways include G2M checkpoint, androgen response, E2F targets and Myc targets as analyzed by GSEA (Supplementary Table 1 and 2). ARVib-7 and ARVib-31 regulated genes were mainly clustered in two major groups when compared with DMSO treatment as plotted by heatmap using hierarchical clustering, indicating a high degree of concordance in the expression changes induced by ARVib-7 and ARVib-31 treatment (Fig. 2B left). Genes that are characterized as AR targets or androgen-induced genes were significantly inhibited by both ARVib-7 and ARVib-31 (Fig. 2B middle). Significantly, both ARVib-7 and ARVib-31 strongly inhibited the expression of genes preferentially upregulated by AR-V7 (Fig. 2B right). Further GSEA analysis revealed that AR and AR-V7 pathways were significantly blocked by ARVib-7 and ARVib-31 treatment in C4-2B MDVR cells. Both ARVib-7 and ARVib-31 robustly disrupted AR and AR-V7 target gene programs (Fig. 2C). qRT-PCR verified that AR and AR-V7 target genes [5, 7], such as KLK2, KLK3, NKX3-1, FKBP5, UBE2C, and Myc were suppressed by both ARVib-7 and ARVib-31 (Fig. 2D). Notably, UBE2C and Myc, which are preferentially upregulated by AR-V7, were significantly suppressed by ARVib-7 and ARVib-31 (Fig. 2D). PSA ELISA results further confirmed that both ARVib-7 and ARVib-31 significantly decreased PSA levels in C4-2B MDVR cells (Fig. 2E). To further examine the effect of ARVib on AR and AR-variant transcriptional activity, C4-2B cells were transiently transfected with AR-V1, AR-V3, AR-V7, AR-V9, and AR-V12 [22–24] (Supplementary Fig. 2A) and then treated with ARVibs or next-generation anti-androgens [Enza, Abi, and apalutamide (Apal)]. ARVib-7, ARVib-31, and next-generation anti-androgens all dramatically inhibited DHT induced AR transcriptional activity. AR-V3, AR-V7, AR-V9, and AR-V12 were constitutively active in CSS conditions in C4-2B cells (Fig. 2F). Their transcriptional activity was only suppressed by ARVib-7 and ARVib-31 but not by next-generation anti-androgens (Fig. 2F). These results indicate ARVib-7 and ARVib-31 disrupt AR/AR-V7 gene programs in Enza-resistant cells. We also determined the effect of ARVib on mutant AR transcriptional activity. Conventional AR antagonists, such as bicalutamide, Enza, and Apal only suppress wild-type AR (WT-AR) transcriptional activity but could not suppress the transcriptional activity mediated by the AR-V7 and AR mutants (T878A, K581R, V716M, and L702H) in FBS conditions [25, 26] (Fig. 2G). In contrast, ARVib-7 and ARVib-31 suppress the transcriptional activity mediated by both WT-AR and mutant AR as well as AR-V7 (Fig. 2G). We performed the same experiments in CS-FBS conditions with or without 10 nM DHT. As shown in Supplementary Fig. 2B, 10 nM DHT successfully activated WT-AR and AR mutants. Enzalutamide and both ARVib-7 and -31 significantly suppressed DHT induced AR activity. The western blot pictures show each AR-variant was successfully transfected into the 293 cells (Supplementary Fig. 2C). Collectively, these results demonstrate that ARVib not only inhibits AR transcriptional program, but also inhibits transcriptional activity mediated by AR variants and AR mutants.

### ARVib inhibits AR/AR-V7 protein expression via HSP70/STUB1-mediated ubiquitin-proteasome system regulation

**Fig. 3:**
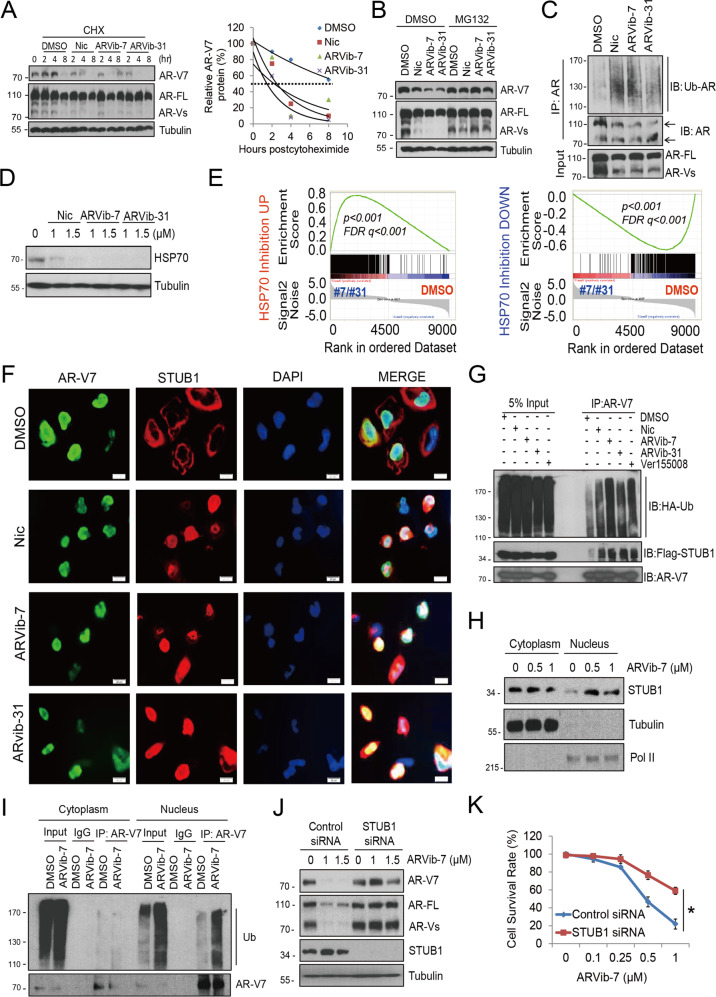
ARVib inhibits AR/AR-V7 protein expression via HSP70/STUB1-mediated ubiquitin-proteasome system regulation. **A** C4-2B MDVR cells were treated with 50 µg/mL cycloheximide with or without ARVibs, after 0, 2, 4, and 8 h, whole-cell lysis was collected and subjected to western blot, half-life of AR-V7 was calculated. **B** C4-2B MDVR cells were treated with 1 μM Nic, ARVib-7 or ARVib-31 for 16 h with 5 μM MG132 for 6 h, total cell lysates were immunoblotted with anti-AR-V7 and AR antibodies. **C** C4-2B MDVR cells were treated with ARVibs, whole-cell lysis was immunoprecipitated with AR antibody and blotted with ubiquitin and AR antibodies. **D** C4-2B MDVR cells were treated with different doses of Nic, ARVib-7, or ARVib-31 for 24 h, whole-cell lysates were collected and subjected to western blot. **E** GSEA demonstrates strong enrichment of the HSP70 inhibition signature in resistant cells treated with ARVibs. The signature was defined by genes with significant expression changes by HSP70 inhibition in prostate cancer cells [27]. **F** 293 cells were co-transfected with AR-V7, HSP70, and Flag-STUB1 for 3 days, and then treated with 1 μM Nic, ARVib-7, or ARVib-31 for 24 h, AR-V7 and STUB1 were visualized by dual immunofluorescence staining. White arrows indicate the typical staining of cells in each group. Nuclei were stained by DAPI. Scale bar 20 µm. **G** AR-V7, HSP70, and Flag-STUB1 were overexpressed in 293 cells, which were then treated with DMSO, Nic, ARVib-7, ARVib-31, or VER for 16 h. Total cell lysates were immunoprecipitated with anti-AR-V7 antibody and immunoblotted with anti-HA-Ub, Flag-STUB1, and AR-V7 antibodies. **H** CWR22Rv1 cells were treated with DMSO, 0.5 or 1.0 μM ARVib-7 for 16 h and 5 μM MG132 for additional 6 h, cytosolic and nuclear proteins were extracted and subjected to western blot. **I** CWR22Rv1 cells were treated with DMSO, 0.5 or 1.0 μM ARVib-7 for 16 h and 5 μM MG132 for additional 6 h, cytosolic and nuclear protein were extracted and immunoprecipitated with AR-V7 antibody and blotted with anti-Ub and AR-V7 antibodies. **J** CWR22Rv1 cells were transiently transfected with STUB1 siRNA for 3 days, and then treated with different doses of ARVib-7 for 16 h. Whole-cell lysates were collected and subjected to western blot. **K** CWR22Rv1 cells were transiently transfected with STUB1 siRNA for 3 days and then treated with different doses of ARVib-7 for 3 days, total cell numbers were determined. *p < *0.05*. Results are the mean of three independent experiments (±S.D.).

We next investigated the underlying mechanisms of AR/AR-V7 suppression by ARVib. We found that neither compound affected mRNA levels of AR/AR-V7 in resistant cells (Supplementary Fig. 3A), suggesting protein level regulation. We then examined the effect of ARVib on AR-V7 protein degradation after new protein synthesis was blocked using cycloheximide. As shown in Fig. 3A and Supplementary Fig. 3B, ARVib-7 and ARVib-31 significantly increased AR-V7 protein degradation compared to the untreated control cells. To examine whether ARVib-7 and ARVib-31 induced AR-V7 protein degradation via the ubiquitin-proteasome system, the 26 S proteasome inhibitor MG132 (5 μM) was added to the cells treated with ARVib. MG132 was able to reduce ARVib-mediated inhibition of AR-V7 protein expression (Fig. 3B and Supplementary Fig. 3C). Furthermore, co-immunoprecipitation assays found that ARVib significantly increased AR and AR-variant ubiquitination, suggesting that ARVib induced AR-V7 degradation is via a proteasome-dependent pathway (Fig. 3C and Supplementary Fig. 3D).

Previous study indicated that the HSP70/STUB1 complex plays a role in AR/AR-V7 regulation. We analyzed RNA-seq data of C4-2B MDVR cells treated with ARVib. Intriguingly, the HSP70 family, which regulates AR-V7 protein homeostasis, was significantly suppressed by ARVib treatment. HSPA1B, one of the major isoforms in regulation of AR/AR-V7 protein stability was the top suppressed gene in the HSP70 family (Supplementary Fig. 4A). We then examined HSP70 suppression by ARVib. As shown in Fig. 3D and Supplemental Fig .4B, ARVib-7 and ARVib-31 are more potent than niclosamide at suppressing HSP70 expression in C4-2B MDVR cells. Further examination by GSEA using the HSP70 inhibition signatures [27] revealed a robust disruption of HSP70 gene programs by ARVib and Nic treatment in Enza-resistant prostate cancer cells (Fig. 3E and Supplementary Fig. 4C). This suggests regulation of specific HSP70 targets by ARVib. Our previous study revealed that STUB1 is decreased in Enza-resistant cells [27], and further nuclear protein extraction suggested that STUB1 expression is significantly decreased in the nucleus but not in the cytoplasm (Supplementary Fig. 4D). HSP70 inhibitors were previously shown to promote STUB1 nuclear translocation and binding to AR-V7 [27]. Here we further confirmed that knockdown of HSP70 in resistant prostate cancer cells significantly promotes STUB1 nuclear translocation by western blot (Supplementary Fig. 4E); suggesting that HSP70 is critical in controlling STUB1 nuclear translocation. To test if ARVib modulates STUB1 nuclear translocation, dual immunofluorescence staining was performed in the 293 cell system. We found that STUB1 and AR-V7 were dissociated in 293 cells when HSP70 was overexpressed. AR-V7 was dominantly present in the nucleus and STUB1 was mostly in the cytoplasm. However, both ARVib-7 and ARVib-31 treatment significantly enhanced AR-V7 and STUB1 colocalization (Fig. 3F and Supplementary Fig. 4F). Co-IP assays determined that ARVib promotes STUB1 binding with AR-V7 and enhances AR-V7 ubiquitination. ARVib significantly increased AR-V7 ubiquitination more than the other niclosamide analogs and the classical HSP70 inhibitor VER155008 (Fig. 3G). These observations were also tested in endogenous protein regulation in CWR22Rv1 and C4-2B MDVR cells. As shown in Fig. 3H and Supplementary Fig. 5A, ARVib-7 treatment significantly promoted STUB1 nuclear translocation and increased AR-V7 ubiquitination in the nucleus (Fig. 3I and Supplementary Fig. 5B) in resistant cells. Knockdown of STUB1 in resistant cells profoundly rescued AR and AR-V7 suppression by ARVib (Fig. 3J and Supplementary Fig. 5C). This suggests that AR/AR-V7 degradation induced by ARVib is mediated by STUB1. Functionally, knockdown of STUB1 ablated ARVib induced growth inhibition in resistant cells (Fig. 3K and Supplementary Fig. 5D). These results suggest that ARVib modulates HSP70/STUB1 machinery and controls AR/AR-V7 protein turnover.

### ARVib suppresses resistant prostate cancer cell growth and induces apoptosis

**Fig. 4:**
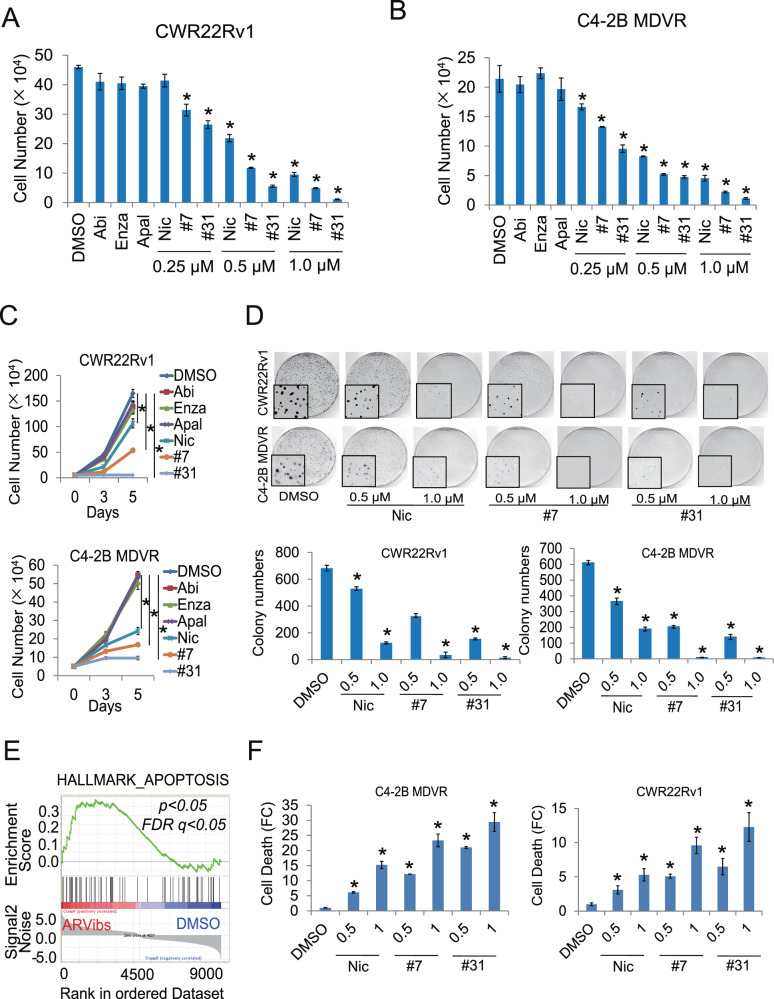
ARVib suppresses resistant prostate cancer cell growth and induces apoptosis. **A, B** CWR22Rv1 and C4-2B MDVR cells were cultured in FBS conditions and treated with DMSO, 5 µM abiraterone, 20 µM enzalutamide, 20 µM apalutamide or different doses (0.25, 0.5, 1.0 µM) of ARVibs for 3 days. Total cell numbers were determined. **C** CWR22Rv1 and C4-2B MDVR cells were cultured in FBS conditions and treated with DMSO, 5 µM abiraterone, 20 µM enzalutamide, 20 µM apalutamide, or 0.5 µM ARVibs. Total cell numbers were determined at 0, 3, and 5 days. **D** CWR22Rv1 and C4-2B MDVR cells were treated with DMSO and different doses (0.5 and 1.0 µM) of ARVibs. The colony numbers were determined. **E** GSEA of the HALLMARK_apoptosis pathway in C4-2B MDVR cells treated with ARVibs, as compared to DMSO. **F** CWR22Rv1 and C4-2B MDVR cells were treated with DMSO and different doses (0.5 and 1.0 µM) of ARVibs. Cell death rate was determined by cell death ELISA kit. *p < *0.05*. Results are the mean of three independent experiments (±S.D.).

We next characterized growth inhibition by ARVib treatment. CWR22Rv1 and C4-2B MDVR cells were treated with Abi, Enza, Apal (ARN), ARVib-7, or ARVib-31 for 3 days, total cell numbers were determined. As shown in Fig. 4A-4B, both C4-2B MDVR and CWR22Rv1 cells are resistant to Abi, Enza, and Apal treatment; however, ARVib-7 and ARVib-31 significantly induced growth inhibition. These results were confirmed at different time points, as shown in Fig. 4C. At 5 days, both ARVib-7 and ARVib-31 strongly suppressed cell growth in both CWR22Rv1 and C4-2B MDVR cells, ARVib-7 and ARVib-31 were more effective than niclosamide treatment in both cell lines. Previous studies revealed that niclosamide suppressed AR negative prostate cancer cell growth [28]; here we showed that both ARVib-7 and ARVib-31 suppressed DU145 and PC3 cells in a dose-dependent manner (Supplementary Fig. 5E). We also performed clonogenic assays to examine colony inhibition by ARVib. As shown in Fig. 4D, both ARVib-7 and ARVib-31 suppressed colony formation in a dose-dependent manner in C4-2B MDVR and CWR22Rv1 cells. GSEA analysis showed that the apoptosis pathway was activated by ARVib-7 and ARVib-31 treatment in C4-2B MDVR cells (Fig. 4E). Cell death ELISA suggested that ARVib-7 and ARVib-31 significantly increased cell death in both C4-2B MDVR and CWR22Rv1 cells (Fig. 4F). ARVib-7 and ARVib-31 were more effective than niclosamide in inducing cell death in resistant cells. These results suggest that ARVib significantly suppresses drug resistant prostate cancer cell growth and induces apoptosis.

### ARVib has improved bioavailability and suppresses advanced prostate tumor growth

**Fig. 5:**
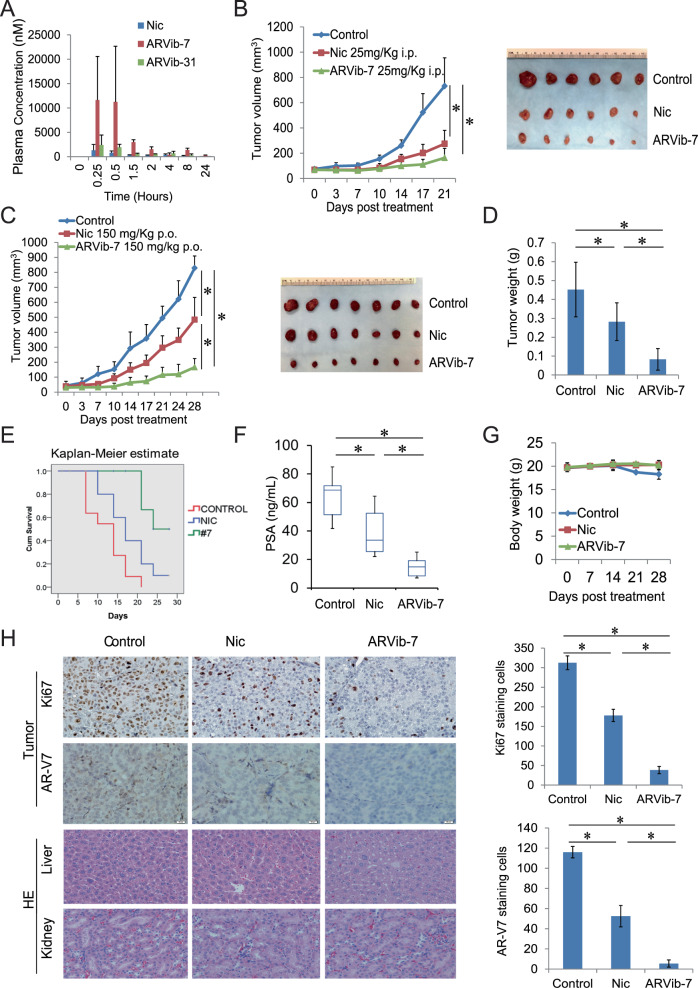
ARVib has improved bioavailability and suppresses advanced prostate tumor growth. **A** 200 mg/Kg niclosamide, ARVib-7 or ARVib-31 were orally administrated to SD rats and rat plasma levels of each drug was determined at different time point. **B** Mice bearing CWR22Rv1 xenografts were treated with vehicle control, niclosamide (25 mg/Kg i.p.), ARVib-7 (25 mg/Kg i.p) for 3 weeks (*n* = 6). Tumor volumes were measured twice weekly. Tumors were photographed and weighed. Scale bar 1 cm. Data represent means ± S.D. from six mice per group. **C** Mice bearing LuCaP 35CR xenografts were treated with vehicle control, niclosamide (150 mg per Kg p.o.), ARVib-7 (150 mg per Kg p.o.) for 4 weeks (*n* = 7). Tumor volumes were measured twice weekly. Scale bar 1 cm. Data represent means ± S.D. from seven mice per group. **D** Tumors were weighed. **E** Kaplan–Meier curves of niclosamide and ARVib-7 treatment in LuCaP 35CR tumors. **F** PSA expression in mice serum was examined in different treatment groups. **G** Body weight was determined. **H** IHC staining of Ki67 and AR-V7 in each group was performed and quantified. H&E staining of kidney and liver from each group was performed. *p < 0.05.

Niclosamide has poor bioavailability in vivo. To determine if ARVib has improved bioavailability, we measured PK parameters in male Sprague-Dawley rat plasma. As shown in Table 1 and Fig. 5A, ARVib-7 and ARVib-31 have better PK parameters than niclosamide when orally administered. ARVib-7 in particular had the best PK parameters. C_max_ and AUC_last_ of ARVib-7 were 6-fold higher than niclosamide, indicating that ARVib has improved bioavailability over parental niclosamide. To determine ARVib-7 effects in vivo, we first used CWR22Rv1 tumor xenografts. Mice bearing tumors were treated with niclosamide (25 mg/Kg) and ARVib-7 (25 mg/Kg) through intraperitoneal (i.p) injection. As shown in Fig. 5B and Supplementary Fig. 6A, ARVib-7 significantly inhibited tumor growth and decreased tumor weight comparable to niclosamide treatment. Treatment did not affect mouse body weight in either niclosamide or ARVib-7 treatment groups (Supplementary Fig. 6B). Both treatments significantly decreased AR/AR-V7 protein expression in tumors (Supplementary Fig. 6C). These results suggest that ARVib is effective at treating resistant xenograft tumor growth.

**Table 1:** PK parameters of ARVib in SD rats’ plasma.

Pharmacokinetic parameters	Nic p.o. (200 mg/Kg)	ARVib-7 p.o (200 mg/Kg)	ARVib-31 p.o (200 mg/Kg)
HL_Lambda_z (min)	328.09 ± 173.05	580.54 ± 86.4	276.51 ± 92.17
T _max_ (min)	25.00 ± 8.66	22.5 ± 10.61	25 ± 8.66
C_max_ (nM)	1556.93 ± 974.31	9038.31 ± 8015.51	2685.82 ± 1796.25
Clast	14.41 ± 10.83	311.67 ± 46.8	15.24 ± 5.73
AUClast	248,249.95 ± 91,856.79	1,439,633.38 ± 89,486.89	382,230.2 ± 220,077.03
AUCINF_obs	170,3577.65 ± 89,354.18	256,881.89 ± 167,532.07	388,512.79 ± 217,228.83
AUC_%Extrap_obs	3.67 ± 3.73	15.35 ± 3.08	2.3 ± 1.85

We next tested ARVib-7 against niclosamide in vivo through oral administration using the LuCaP 35CR PDX model, which has AR-V7 overexpression [27, 29]. Our data show that compared to vehicle control, oral administration of niclosamide (150 mg/Kg) suppressed PCa growth by 50% while the same dose of ARVib-7 suppressed tumor volume by ~85% (Fig. 5C). ARVib-7 treated tumor weight was also significantly lower than that of niclosamide treated animals (Fig. 5D). Survival was improved in the ARVib-7 groups compared to the vehicle or niclosamide treated groups as well (Fig. 5E). Niclosamide treatment slightly suppressed tumor PSA expression and ARVib-7 treatment significantly suppressed PSA (Fig. 5F). Treatments did not affect mouse body weights (Fig. 5G). Vital organs, such as liver and kidney, were harvested for histopathologic examination and no significant pathological changes were found in the organs from any group. As shown in Fig. 5H and Supplementary Fig. 6D, livers did not show any vacuolar changes; and there was no sign of inflammation at the renal pelvis in single or combination treatment groups. Immunohistochemical staining of AR-V7 and Ki67 showed that AR-V7 expression and cell proliferation were significantly inhibited by ARVib-7 treatment (Fig. 5H, right). Taken together, these data demonstrate that ARVib-7 has a superior PK profile to that of niclosamide and is highly effective for the treatment of resistant prostate cancer tumor in vivo.

### ARVib improves Enza treatment in vitro and in vivo

**Fig. 6:**
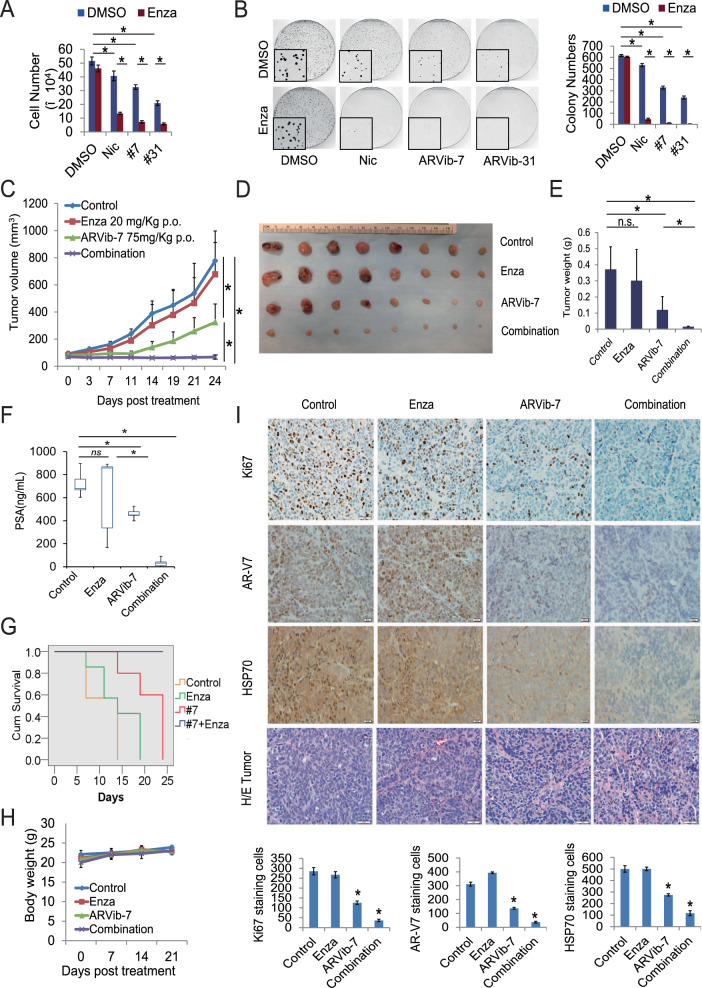
ARVib improves enzalutamide treatment in vitro and in vivo. **A, B** CWR22Rv1 cells were treated with DMSO, 20 µM enzalutamide, 0.5 µM ARVibs or their combination. Cell growth was determined at 3 days and colony formation ability was examined by clonogenic assay. **C** Mice bearing VCaP xenografts were castrated and the relapsed tumors were treated with vehicle control, enzalutamide (25 mg per Kg p.o), ARVib-7 (75 mg per Kg p.o.) or their combination for 24 days (*n* = 8). Tumor volumes were measured twice weekly. **D** Tumors were photographed. Scale bar 1 cm. Data represent means ± S.D. from eight mice per group. **E** Tumors weight. **F** PSA expression in mice serum was examined. **G** Kaplan–Meier curves showing survival benefits of ARVib-7 single treatment, ARVib-7 and enzalutamide combination treatment in relapsed VCaP tumors. **H** Body weight was determined. **I** IHC staining of Ki67, AR-V7, and HSP70 in each group was performed and quantified. *p < 0.05.

Our preliminary screening has determined the ARVib synergistically enhance anti-androgen treatment. Here, using the resistant CWR22Rv1 and C4-2B MDVR cells, we further tested ARVib in combination with Enza in vitro. As shown in Fig. 6A and Supplementary Fig. 6E, Enza had no effect on cell growth in vitro. Both ARVib-7 and ARVib-31 as well as Nic profoundly enhanced Enza treatment in CWR22Rv1 and C4-2B MDVR cells. These results were confirmed by clonogenic assay (Fig. 6B and Supplementary Fig. 6F-G). We then used the relapsed VCaP tumor model to validate the efficacy of combination treatment in vivo. As shown in Fig. 6C-6E, Enza treatment alone insignificantly inhibited tumor growth and produced a tumor growth curve very similar to the untreated control. ARVib-7 (75 mg/Kg orally) alone significantly suppressed tumor growth. Combination of ARVib-7 with Enza further suppressed tumor growth in vivo. Enza treatment slightly, but insignificantly, suppressed tumor PSA expression (*p* = 0.28), ARVib-7 treatment significantly suppressed PSA (*p* = 0.029) and the combination treatment further reduced PSA levels (*p* = 0.0053) (Fig. 6F). Survival was improved in the ARVib-7 group compared to the vehicle or Enza treated groups, and was further extended in the combination treatment group (Fig. 6G). Treatments did not affect mouse body weight (Fig. 6H). Immunohistochemical staining showed that expression of Ki67, AR-V7, and HSP70 was significantly inhibited by ARVib-7 and further reduced by the combination treatment (Fig. 6I). Collectively, the results suggest that ARVib suppresses prostate tumor growth and improves Enza treatment.

## Discussion

In the present study, we identified a new AR/AR-V7 degrader called ARVib from a library of synthetic analogs of niclosamide, which was previously identified as a potent inhibitor of AR and AR-V7. We demonstrate that ARVib has better bioavailability and PK parameters than niclosamide, and can effectively degrade AR/AR-V7 and attenuate AR/AR-V7 downstream target gene expression in prostate cancer cells. Mechanistically, ARVib degrades AR/AR-V7 protein expression through the ubiquitin-proteasome pathway mediated by modulation of HSP70/STUB1 machinery. In addition, ARVib significantly inhibited resistant prostate tumor growth and improved Enza treatment in vitro and in vivo.

Large efforts have been placed in order to identify potential inhibitors of AR-V7. ASC-J9® was developed as an AR degradation enhancer, and has shown efficacy in preclinical studies in slowing progression of Enza-resistant prostate cancer [30]. PROTAC-based BET protein degradation was employed to abolish AR signaling and AR protein level in CPRC mouse xenografts [31]. Most recently, ARD-61 was identified by the PROTAC technology as a potent AR degrader and functions to overcome resistance to enzalutamide treatment without inducing AR-V7 protein degradation [32]. Several small molecule-degraders of full-length AR and AR-V7 are identified to have effective inhibition of AR transcriptional activity and inhibition of resistant cancer cell proliferation [33]. In addition, initially designed as CYP17 lysase inhibitors, Galeterone and VNPT55, also have been demonstrated to significantly downregulate AR splice variants via activation of proteasome degradation [34]. We have previously demonstrated that niclosamide inhibits AR-V7 protein expression and enhances Enza and Abi treatment. However, undesirable pharmacokinetic parameters with poor solubility (0.23 μg/mL) and poor bioavailability (~10%) limit its use in the clinic [20, 21]. In order to take advantage of the efficacy of niclosamide while optimizing its pharmacokinetic features, we synthesized a library of analogs and identified ARVib as new degraders of AR/AR-V7 protein. The newly synthesized ARVib shows superior to niclosamide in bioavailability and achieve better anti-tumor effects in advanced prostate cancer.

Niclosamide initially was discovered as a treatment for tapeworm infection and functions by inducing mitochondrial uncoupling which causes tapeworm energy deficiency. However, its mechanism of action in human cancer cells remains unclear. Niclosamide has been reported to interfere with multiple signaling pathways, including mTORC1, IL-6/JAK/STAT3, ERK, Src, and Wnt signaling in different lineages of cancer cells [35]. The ARVib studied in the current study have a similar chemical structure to niclosamide. Thus, it is possible that they also have ability to suppress these oncogenic pathways in prostate cancer cells. Particularly, niclosamide was reported to have pH-dependent toxicity in AR negative neuroendocrine prostate cancer cells as well as AR negative PC3 cells [28]. In the current study, we discovered that ARVib modulated the HSP70/STUB1 complex and induced AR/AR-V7 protein degradation. As a co-chaperone protein of STUB1, HSP70 assists protein folding and maturation of multiple oncogenes, such as AR and GR [36, 37]. These findings suggest that niclosamide and its analogs may act on several other protein targets in addition to AR and AR-V7.

Cumulative evidence shows that deficiency of proteostasis promotes tumorigenesis, which frequently causes abnormal aggregation of oncogenic proteins, such as c-myc, p53, BCL-2, etc [12, 38–40]. Targeting oncogenic proteins based on their expression of E3 ligase, which is differentially expressed in tumor cells, has become a novel strategy to treat cancer diseases. PROTAC (proteolysis-targeting chimera) links the target proteins to the E3 ligase, promoting protein degradation by the ubiquitin-proteasome system [41]. Similarly, chemical inducers of proximity (CIPs)-based techniques are employed to target chimeric E3 ligase-complexes by modulating the proteasome system, inducing apoptosis of tumor cells and oncogenic proteins [42]. The current study reiterates the vital role of imbalanced proteolysis in CRPC. Niclosamide analogs, in particular ARVib-7 and ARVib-31, direct the nuclear translocation of E3 ubiquitin ligase STUB1 to bind to AR-V7, leading to AR-V7 ubiquitination and degradation. ARVib-7 and ARVib-31, exhibited profound AR-V7 inhibitory effects by activation of STUB1/HSP70 machinery-mediated proteolysis (Fig. 3G).

Targeting the ubiquitin-proteolysis pathway to degrade oncogenic proteins has been emerging as an attractive strategy to treat cancer [14, 43]. Various drugs targeting the proteolysis pathway are under development, including the first FDA-approved proteasome inhibitor Bortezomib, which is used to treat multiple myeloma [44] and the SP90 inhibitor 17-AAG is in clinical trial for various cancer types [45]. Our previous study investigated HSP70/STUB1 and determined HSP70 formed a complex with STUB1 to promote AR/AR-V7 protein stabilization and degradation. HSP70 inhibition promotes AR/AR-V7 degradation via STUB1 binding and reverses anti-androgen resistance [27]. We have identified a potential mechanism of ARVib-mediated AR/AR-V7 protein degradation. ARVib degrades AR/AR-V7 protein expression through the ubiquitin-proteasome pathway mediated by HSP70/STUB1 machinery modulation. ARVib suppresses HSP70 expression and promotes STUB1 nuclear translocation, where STUB1 binds to AR/AR-V7 and promotes its ubiquitination and degradation. Emerging evidence suggests that HSP70 is elevated in various tumors [46]. Like other HSPs family members, HSP70 plays an indispensable role in protein folding and maturation and also acts as an oncogenic protein, protecting cancer cells from apoptosis through binding to pro-apoptotic proteins and suppressing pro-apoptotic kinases [47, 48]. In prostate cancer, HSP70 is a biomarker for patient prognosis and survival since overexpression of HSP70 is associated with tumorigenesis and drug resistance [49]. HSP70 inhibition has been shown to decrease full-length AR and AR-variants protein levels and transcriptional activity, while concomitantly degrading other oncogenic proteins [37, 50].

In conclusions, we have identified a new AR/AR-V7 degrader named ARVib and showed that ARVib effectively degrades AR/AR-V7 protein through the ubiquitin-proteasome pathway mediated by the HSP70/STUB1 complex and attenuates AR/AR-V7 downstream target gene expression in prostate cancer cells. ARVib significantly inhibits resistant prostate tumor growth and improves Enza treatment in vitro and in vivo, suggesting a clinical potential for development as an AR/AR-V7 degrader to treat resistant CRPC.

## Materials and methods

### Cells lines and tissue culture

C4-2B and CWR22Rv1 cells were maintained in RPMI 1640 supplemented with 10% fetal bovine serum (FBS), 100 units per ml penicillin and 0.1 mg per ml streptomycin. 293 and VCaP cells were maintained in DMEM supplemented with 10% fetal bovine serum (FBS), 100 units per ml penicillin and 0.1 mg per ml streptomycin. All experiments with cell lines were performed within 6 months of receipt from the American Type Culture Collection (ATCC) or resuscitation from cryopreservation. C4-2B cells were kindly provided and authenticated by Dr. Leland Chung, Cedars-Sinai Medical Center (Los Angeles, CA). The resistant cells are referred to as C4-2B MDVR (C4-2B enzalutamide resistant) [51]. C4-2B MDVR cells were maintained in 20 μM Enza containing medium. Parental C4-2B cells were passaged alongside the resistant cells as an appropriate control. All cell lines have been routinely tested for mycoplasma by PCR and authenticated by the short tandem repeat (STR) method. All cells were maintained at 37 °C in a humidified incubator with 5% carbon dioxide. Enza and Ver155008 (VER) were purchased from Selleck Chemicals.

### Plasmids and cell transfection

For small interfering RNA (siRNA) transfection, cells were seeded at a density of 0.5 × 10^5^ cells per well in 12-well plates or 2 × 10^5^ cells per well in six-well plates and transfected with 20 nM of siRNA (Invitrogen) targeting the HSP70 sequence (HSPA1A/HSPA1B, Catalog# 262305 and 262306), STUB1 sequence (Catalog# 215046) or control siRNA (Catalog# 12935300) using Lipofectamine-iMAX (Invitrogen). The effect of siRNA-mediated gene silencing was examined using qRT-PCR and western blot 2–3 days after transfection. Cells were transiently transfected by expressing plasmids for vectors, AR-FL, AR-V1, AR-V3, AR-V7, AR-V9, AR-V12 (AR-V567es), wild-type AR, AR mutants (T878A, K581R, L702H, V716M), Flag-STUB1, HA-Ubiquitin or HSP70 (HSPA1B, OriGene, Catalog# SC116767) using Lipofectamine 2000 (Invitrogen).

### Protein extraction and western blotting

Whole-cell protein extracts were incubated for 16 h at 4^o^C with the indicated primary antibodies AR (441), AR (N20), AR (C-19), HSP70 (F-3 and H-300), STUB1(H231 and G-2), HA (F-3), Ubiquitin (P4D1 and FL76), 1:1000 dilution, Santa Cruz Biotechnology, Santa Cruz, CA; STUB1 (C3B6, 1:100 for IP, Cell Signaling antibody); AR-V7 (AG10008, Mouse monoclonal antibody, 1:1000 dilution, precision antibody); FLAG® M2 monoclonal antibody (F1804, 1:1000 dilution for western blot, 1:200 for IP, Sigma-Aldrich, St. Louis, MO); Tubulin (T5168, Monoclonal Anti-α-Tubulin antibody, 1:5000 dilution, Sigma-Aldrich, St. Louis, MO). Following secondary antibody incubation, immunoreactive proteins were visualized with an enhanced chemiluminescence detection system (Millipore, Billerica, MA). The bands were quantified by ImageJ.

### Luciferase reporter assay

C4-2B cells were plated in 12-well plates (1 × 10^5^) and grown to 80% confluence and transiently transfected using Lipofectamine 2000 (Invitrogen). pGL3-PSA6.0 luciferase construct was co-transfected with pRL-TK (TK promoter-Renilla luciferase construct as internal control). Briefly, pGL3-PSA6.0 luciferase construct and pRL-TK along with WT-AR, AR variants or HSP70 were mixed and transfected. Cells were then treated with anti-androgens or ARVib-7 or ARVib-31 with or without DHT. The luciferase activity was determined 24 h after treatment using a dual-luciferase reporter assay system (Promega). Cell lysates (35 μL per well) were used for measurement of luciferase activity in a luminometer by first mixing the cell lysates (25 μL) with 20 μL luciferase assay reagent for measuring firefly luciferase activity and subsequently adding 20 μL Stop-Glo reagent for measuring Renilla luciferase activity. Data were normalized to Renilla luciferase activity.

### Cell growth assay

C4-2B MDVR and CWR22Rv1 cells were seeded on 12-well plates at a density of 0.5 × 10^5^ cells/well in RPMI 1640 media containing 10% FBS and treated with anti-androgens, ARVib-7 or ARVib-31 for 3 or 5 days. Total cell numbers were counted and the cell survival rate (%) was calculated. Cell survival rate (%) = (Treatment group cell number/Control group cell number) ×100%. CWR22Rv1 cells, C4-2B MDVR cells were seeded on 12-well plates at a density of 0.5 × 10^5^ cells/well in RPMI 1640 media containing 10% FBS and treated with 0.25 or 0.5 μM ARVibs with or without 20 μM Enza in media containing FBS. Total cell numbers were determined after 3 days. The coefficient of drug interaction (CDI) [52] is calculated as follows: CDI = AB/(A × B). Wherein A is the growth inhibition effect of anti-androgen (enzalutamide or abiraterone) treatment, B is growth inhibition effect of ARVibs treatment and AB is the growth inhibition effect of combination treatment. The CDIs were analyzed to determine the synergism of the two drugs in combination (A CDI value <1, =1, or >1 indicates that the drugs are synergistic, additive or antagonistic respectively).

### Clonogenic assay

C4-2B MDVR or CWR22Rv1 cells were treated with different doses of ARVib-7 or ARVib-31 in media containing 10% complete FBS with or without 20 μM Enza. Cells were plated at equal density (1000 cells/dish) in 100 mm dishes for 14 days; the medium was changed every 7 days. The colonies were rinsed with PBS before staining with 0.5% crystal violet/4% formaldehyde for 30 min and the number of colonies was counted.

### Cell death ELISA

C4-2B MDVR cells were seeded on 12-well plates (1 × 10^5^ cells/well) in RPMI 1640 media containing 10% FBS and treated with DMSO or different doses of ARVib or Nic#31 for 3 days. Mono- and oligonucleosomes in the cytoplasmic fraction were measured by Cell Death Detection ELISA kit (Roche, Cat. NO. 11544675001) according to the manufacturer’s instructions. Briefly, floating and attached cells were collected and homogenized in 400 µL of incubation buffer. The wells were coated with antihistone antibodies and incubated with the lysates, horseradish peroxidase–conjugated anti-DNA antibodies, and the substrate. Absorbance was measured at 405 nm.

#### Real-time quantitative RT-PCR

Total RNAs were extracted using TriZOL reagent (Invitrogen). cDNAs were prepared after digestion with RNase-free RQ1 DNase (Promega). The cDNAs were subjected to real-time reverse transcription-PCR (RT-PCR) using Sso Fast Eva Green Supermix (Bio-Rad) according to the manufacturer’s instructions and as described previously [51]. Each reaction was normalized by co-amplification of actin. Triplicates of samples were run on default settings of Bio-Rad CFX-96 real-time cycler. The primer sequences are listed in Supplementary data.

#### Co-immunoprecipitation assay

Equal amounts of cell lysates (1500 µg) were immunoprecipitated using 1 µg of Flag antibody, HSP70 antibody, AR-V7 antibody, AR (N20) antibody or STUB1 antibody with 50 µL of protein A/G agarose with constant rotation 16 h. The immunoprecipitants were washed with 10 mM HEPES (pH 7.9), 1 mM EDTA, 150 mM NaCl, and 1% Nonidet P-40 twice with 1 mL each. The precipitated proteins were eluted with 30 µL of SDS-PAGE sample buffer by boiling for 10 min. The eluted proteins were electrophoresed on 8% SDS-PAGE, transferred to nitrocellulose membranes, and probed with indicated antibodies.

### Dual immunofluorescence assay

1 × 10^4^ 293 cells were plated in four-well Nunc™ Lab-Tek™ II Chamber Slides and transfected with AR-V7, HSP70 and Flag-STUB1 for 3 days and then treated with ARVib-7 or ARVib-31 with 5 µM MG132 for 16 h. Cells were fixed with 4% paraformaldehyde, permeabilized with 0.5% Triton X-100, and incubated with 1% BSA to block nonspecific binding. Cells were incubated with anti-AR (N20, Santa Cruz Biotechnology) and anti-Flag antibodies (Sigma) overnight. Intracellular AR-V7 was visualized with FITC-conjugated secondary antibodies, Flag-STUB1 was visualized with Texas red conjugated secondary antibodies and nuclei were visualized with DAPI by All-in-One Fluorescence Microscope (BZ-X700).

### RNA-sequencing data analysis

C4-2B MDVR cells were treated with vehicle, or 1.5 μM ARVib-7, or ARVib-31 for 24 h before RNA extraction. Indexed RNA-seq libraries were prepared from total RNA (1 μg) using the KAPA Stranded mRNA-Seq Library Kit (Kapa Biosystems), according to the manufacturer’s instructions. Subsequently, libraries were combined for multiplex sequencing on an Illumina HiSeq 4000 System (2 × 150 bp, paired-end, ~30 × 10^6^ reads per sample). Data analysis was performed with a HISAT-StringTie-Cuffnorm pipeline for mapping/alignment of raw sequence reads (FASTQ format) to the reference human genome assembly (GRCh38/hg38), transcript assembly, and quantitation of gene and transcript expression as FPKM (Fragments Per Kilobase of transcript per Million mapped reads). Data were annotated for 60,658 unique genes/transcripts with GENCODE, Release 26 (GRCh38.p10). Principal component analysis (PCA) was conducted on the FPKM gene-level data for all genes/transcripts passing filter (Filtered on Expression > 0.1) in the raw data (GEO accession number: GSE158556). The relatedness of the differentially expressed genes from treatment with different ARVibs was depicted with a Venn diagram. Expression patterns of genes commonly regulated by ARVib treatment were examined by hierarchical clustering analysis with StrandNGS software (Strand Life Sciences).

### Geneset enrichment analysis (GSEA)

GSEA was performed using the Java desktop software (http://software.broadinstitute.org/gsea/index.jsp) [53]. Genes were ranked according to the shrunken limma log2 fold changes, and the GSEA tool was used in ‘pre-ranked’ mode with all default parameters. KEGG-Ubiquitin mediated proteolysis pathway was used in the GSEA analysis.

### Pharmacokinetic analysis

Blood samples (around 50 µl) from SD rats were collected from the tail veins punctured by a lancet at 0, 15 mins, 30 mins, 60 mins, 2 h, 4 h, 8 h, and 24 h. To quantify niclosamide, ARVib-7 and ARVib-31 concentrations in the plasma samples, rat plasma standards at 0, 0.5, 1, 5, 10, 50, 100, 500, and 1000 ng/ml for each drug were made for establishing a rat plasma calibration curve. All calibrators were prepared in blank rat plasma with lithium heparin as the anticoagulant (Bioreclamation IVT, Westbury, NY). ARVib-31 plasma calibration curve could not be established as this was converted to niclosamide in the blank rat plasma immediately after spiking. Hundred microliters of the resulting rat plasma standard calibrators and rat plasma samples were loaded into each well of a 96-well Isolute PLD+ protein and phospholipid removal plate (Biotage, Uppsala, Sweden). One hundred eighty microliters of the internal standard (IS), 13C6-niclosamide (Millipore Sigma, St. Louis, MO), 100 ng/ml in acetonitrile (ACN, Fisher Scientific, Waltham, MA), was then added to each well. Five microliters of the filtrate was injected into a Waters (Milford, MA) Acquity UPLC with a BEH C18 1.7 µm, 2.1 mm × 50 mm column. To run the UPLC, H_2_O with 0.1% (v/v) of formic acid (both from Fisher Scientific, Waltham, MA) was used as mobile phase A (A) and ACN with 0.1% of formic acid was used as mobile phase B (B). The following UPLC gradient program was used for the separation: 0–1.5 min, 10% B; 1.51–3.5 min, 95% B; 3.51–5 min, 10% B. The output of the UPLC was fed to a Waters Xevo TQ-S triple quadrupole MS/MS system, which was used to ionize target molecules with the ESI- probe and monitor the ion m/z fragmentation transitions from 325 → 171 for niclosamide quantification, 348 → 194 for ARVib-7 quantification, and 331 → 177 for 13C6-niclosamide quantification at multiple reaction monitoring (MRM) mode. The calibration curve was fitted with weighted (1/x2) least-squares linear regression algorithm. The extraction yield was 58.29 ± 9.45% for niclosamide and 84.45 ± 15.24% for ARVib-7. The matrix effect enhanced the niclosamide MS signal by 25.16% and inhibited ARVib-7 MS signal by 3.8%. Both inter- and intra-batch accuracy of all levels of QC samples were within 0 to 11% (%deviation) and both intra- and inter-batch precision of all levels of QC samples were also within 0 to 11% (%CV).

### Animal studies and treatment regimens

All animals used in this study received humane care in compliance with applicable regulations, policies, and guidelines relating to animals. All experimental procedures using animals were approved by the Institutional Animal Care and Use Committee of UC Davis. CWR22Rv1 cells (3 million) were mixed with matrigel (1:1) and injected subcutaneously into the flanks of 4-5 week old male C.B17/lcrHsd-Prkdc-SCID mice (ENVIGO). Tumor-bearing mice (tumor volume around 50–100 mm3) were randomized into six groups (7 mice per group) and treated as follows: (1) vehicle control (15% Cremophor EL, 82.5% PBS and 2.5% dimethyl sulfoxide (DMSO), intraperitoneal (i.p.)), (2) niclosamide (25 mg per Kg i.p.), (3) ARVib (25 mg per kg, i.p.). Tumors were measured using calipers twice a week and tumor volumes were calculated using length × width × width × 0.52. Tumor tissues, liver, and kidney were harvested and weighed after 3 weeks of treatment. Tumor tissues, liver and Kidney were paraffin embedded and H/E stained.

To assess the effect of oral ARVib administration on the growth of PDX tumors, the LuCaP35 CR model was obtained from the University of Washington and established in the UC Davis Cancer Center. Briefly, 3-4 weeks C.B17/lcrHsd-Prkdc-SCID mice (ENVIGO) were surgically castrated. Two weeks later, ∼20- to 30-mm^3^ pieces of LuCaP 35CR tumor were implanted into the precastrated SCID mice. When tumors reached 50–100 mm^3^, mice were randomized into four groups (six mice per group) and treated as follows: (1) vehicle control (0.5% weight/volume (w/v) Methocel A4M p.o.), (2) niclosamide (150 mg per kg, p.o.), (3) ARVib (150 mg per kg, p.o.). Tumors were measured using calipers twice a week and tumor volumes were calculated using length × width × width × 0.52. Tumor tissues were harvested and weighed after 5 weeks of treatment. Serum was collected for PSA determination.

To assess the combination effects of ARVib and Enza in vivo, VCaP cells (4 × 10^6^) were mixed with matrigel (1:1) and injected subcutaneously into the flanks of 3–4 week male SCID mice. After PSA levels are detectable (5–10 ng/mL), mice were castrated and the tumor volume was monitored, after the tumor grew back to 50–100 mm^3^, tumor-bearing mice were randomized into three groups (five mice in each group) and treated as follows: (1) vehicle control (0.5% weight/volume (w/v) Methocel A4M p.o.), (2) Enzalutamide (20 mg/Kg p.o.), (3) ARVib (75 mg/kg, p.o.), (4) Combination. Tumors were measured using calipers twice a week and tumor volumes were calculated using length × width × width × 0.52. Tumor tissues were harvested after 3 weeks of treatment.

### Measurement of mouse serum PSA

Blood from the LuCaP 35CR and relapsed VCaP tumor model mice was collected and the serum was isolated. PSA levels were measured using a PSA ELISA Kit (United Biotech, Inc., Mountain View, CA) according to the manufacturer’s instructions.

### Immunohistochemistry

Tumors were fixed by formalin and paraffin embedded tissue blocks were dewaxed, rehydrated, and blocked for endogenous peroxidase activity. Slides were incubated with anti-HSP70 (F-3, at 1:300; Santa Cruz), anti-Ki67 (at 1:500; Neomarker), or anti-AR-V7 (at 1:200; Precision) at 4 °C overnight. Slides were then washed and incubated with biotin-conjugated secondary antibodies for 30 min, followed by incubation with avidin DH-biotinylated horseradish peroxidase complex for 30 min (Vectastain ABC Elite Kit, Vector Laboratories). The sections were developed with the diaminobenzidine substrate kit (Vector Laboratories) and counterstained with hematoxylin. Nuclear staining of cells was scored and counted in 5 different vision fields. Images were taken with an Olympus BX51 microscope equipped with DP72 camera.

### Statistical analysis

Statistical analyses were performed with SPSS16.0. Raw data are summarized by means, standard deviations (SD), and graphical summaries and transformed if necessary to achieve normality. Data from the in vitro experiments are presented as means ± SD from three independent experiments. Differences between individual groups were analyzed by two-tailed Student’s *t*-tests for single comparisons or one-way analysis of variance (ANOVA) followed by the Scheffé procedure for multiple group comparisons. In the tumor growth experiments, size of the tumor at sacrifice serves as the primary response measure. Tumor growth and PSA across groups was analyzed by ANOVA. *p* < 0.05 was considered statistically significant.

## Supplementary information

supplementary figures and tables
