# Supplementary material for: ARVib suppresses growth of advanced prostate cancer via inhibition of androgen receptor signaling
Source: Oncogene. 2021 Jul 16;40(35):5379–92. doi: 10.1038/s41388-021-01914-2 (PMC8413131; doi:10.1038/s41388-021-01914-2)
Supplement: Supplementary file 1 — supplementary figures and tables [file 41388_2021_1914_MOESM1_ESM.pdf]

Supplementary Information

ARVib suppresses growth of advanced prostate cancer via inhibition of androgen receptor signaling

Liu, et al.

Supplementary Figure 1

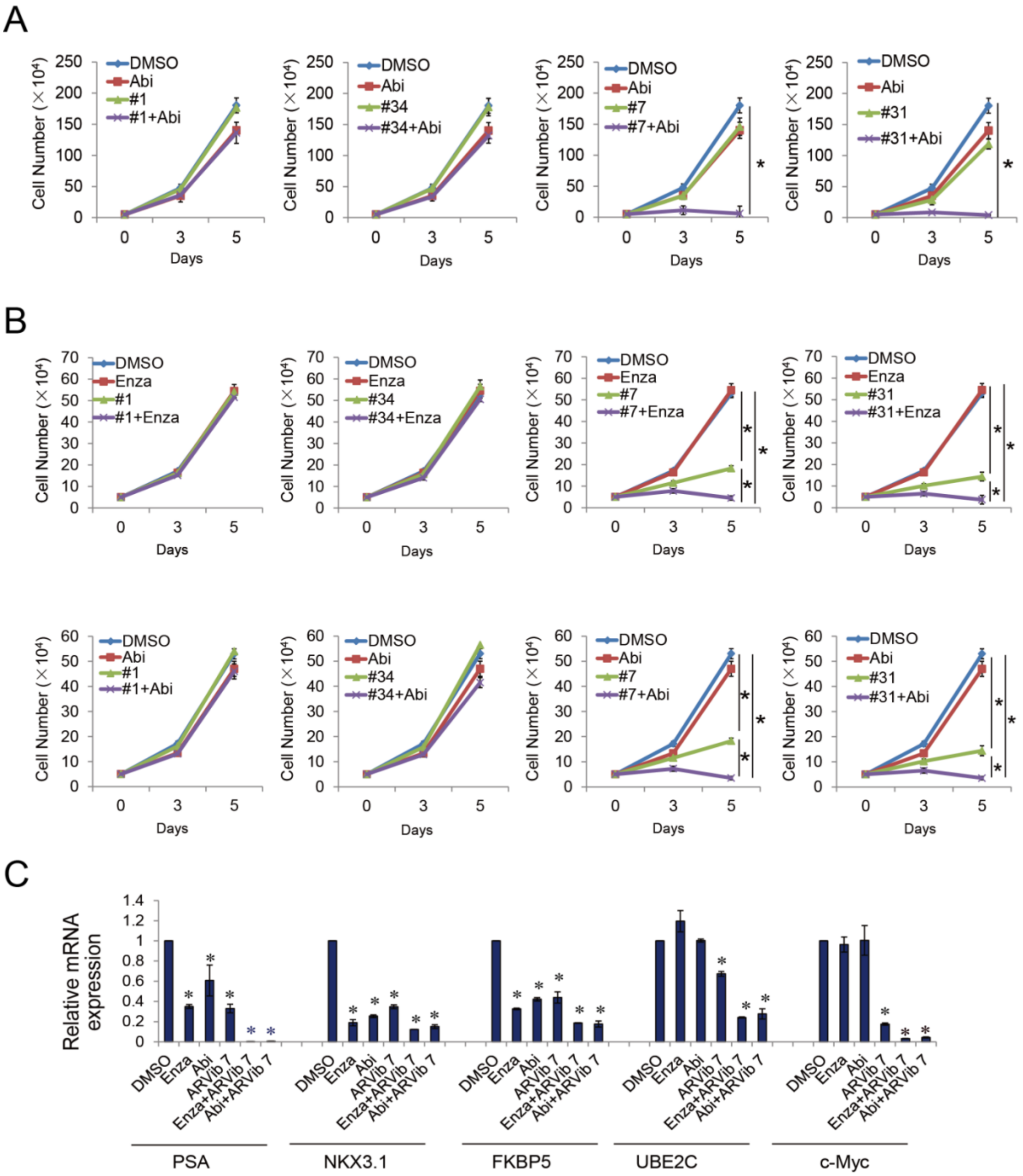

Supplementary Figure 1. Identification of potent ARVib in drug resistant prostate cancer cells. (A) CWR22Rv1 cells were treated with 0.25 μM ARVib-1, ARVib-34, ARVib-7 or ARVib-31 in combination with 5μM abiraterone acetate for 3 days. Total cell numbers were determined at 0, 3 and 5 days. **(B)** C4-2B MDVR cells were treated with 0.25 μM ARVib-1, ARVib-34, ARVib-7 or ARVib-31 in combination with 20 μM enzalutamide or 5 μM abiraterone acetate. Total cell numbers were determined at 0, 3 and 5 days. **(C)** C4-2B MDVR cells were treated with 0.25 μM ARVib-7 with or without Enza/Abi for 5 days, total RNA was extracted and the mRNA expression of AR/AR-V7 target genes were examined by qRT-PCR. * $p<0.05$. Results are the mean of three independent experiments (±S.D.). Statistical analysis was performed using two tailed Student's t test.

Supplementary Figure 2

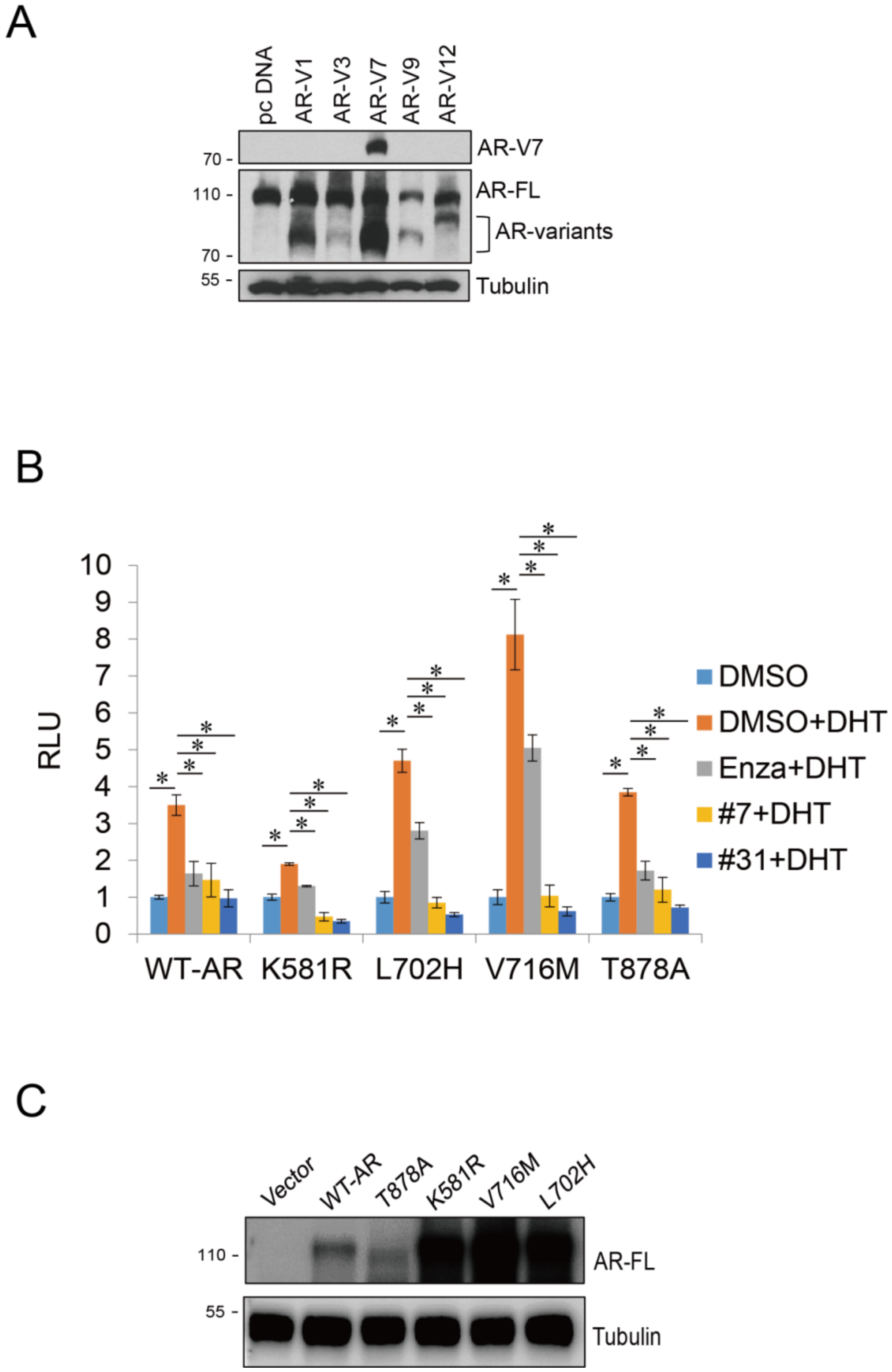

Supplementary Figure 2. ARVib suppress AR variants and mutant AR activity. (A) C4-2B cells were transiently transfected with pcDNA or different AR variants for 3 days, whole cell lysates were collected for western blot. **(B)** 293 cells were transiently transfected with vector, AR-V7, AR mutants plus PSA promoter luciferase plasmids in CS-FBS condition, and then treated with 20 μM enzalutamide or 1 μM ARVibs with 10 nM DHT for 16 hours. PSA luciferase activity was determined. **(C)** The AR mutants transfected in 293 cells were examined by western blot. * $p<0.05$. Results are the mean of three independent experiments (±S.D.).

Supplementary Figure 3

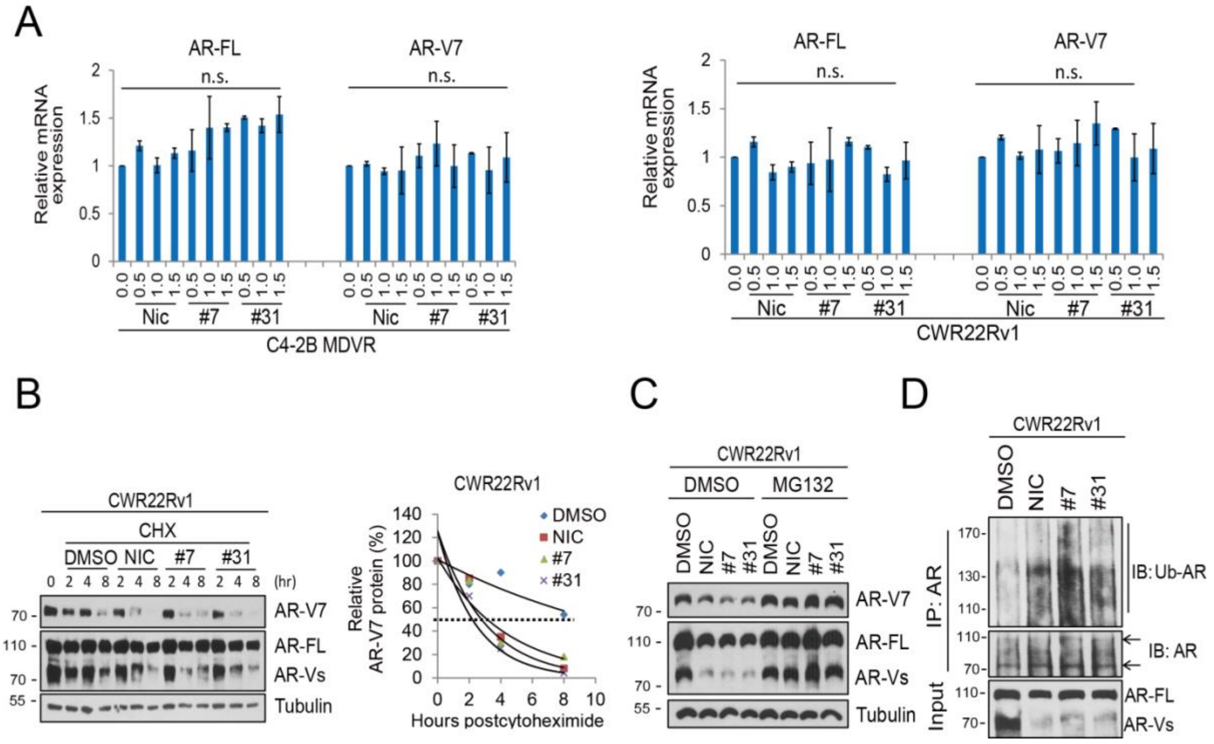

Supplementary Figure 3. ARVib degrades AR/AR-V7 via regulation of the ubiquitin proteasome system. (A) C4-2B MDVR and CWR22Rv1 cells were treated with different doses of ARVib 16 hours, total RNA was extracted and mRNA levels of AR-V7 and full-length AR were examined by real-time PCR. **(B)** CWR22Rv1 cells were treated with 50 µg/mL cycloheximide with or without ARVib. After 0, 2, 4 and 8 hours, whole cell lysates were collected and subjected to western blot and the half-life of AR-V7 was calculated. **(C)** CWR22Rv1 cells were treated with DMSO or 1µM ARVib 16 hours and then treated with 5 µg/mL MG132 for additional 6 hours. Whole cell lysates were collected and subjected to western blot. (**D**). CWR22Rv1 cells were treated with DMSO or 1µM ARVib 16 hours. Whole cell lysates were collected and immunoprecipitated with AR antibody and blotted with anti-Ub and AR antibodies.

Supplementary Figure 4

A

	Genes	ARVib-7	ARVib-31
HSP70 FAMILY	HSPA1B	-2.5721	-4.1687
	HSPA4	-1.4239	-1.6279
	HSPA14	-1.2818	-1.1737
	HSPA4L	-1.3108	-1.2232

B

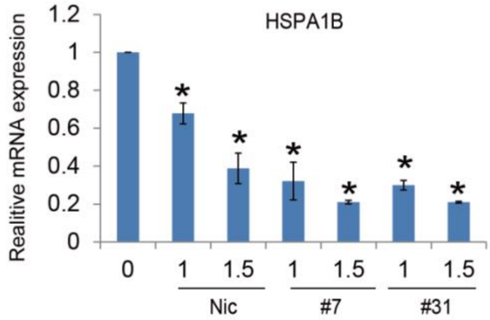

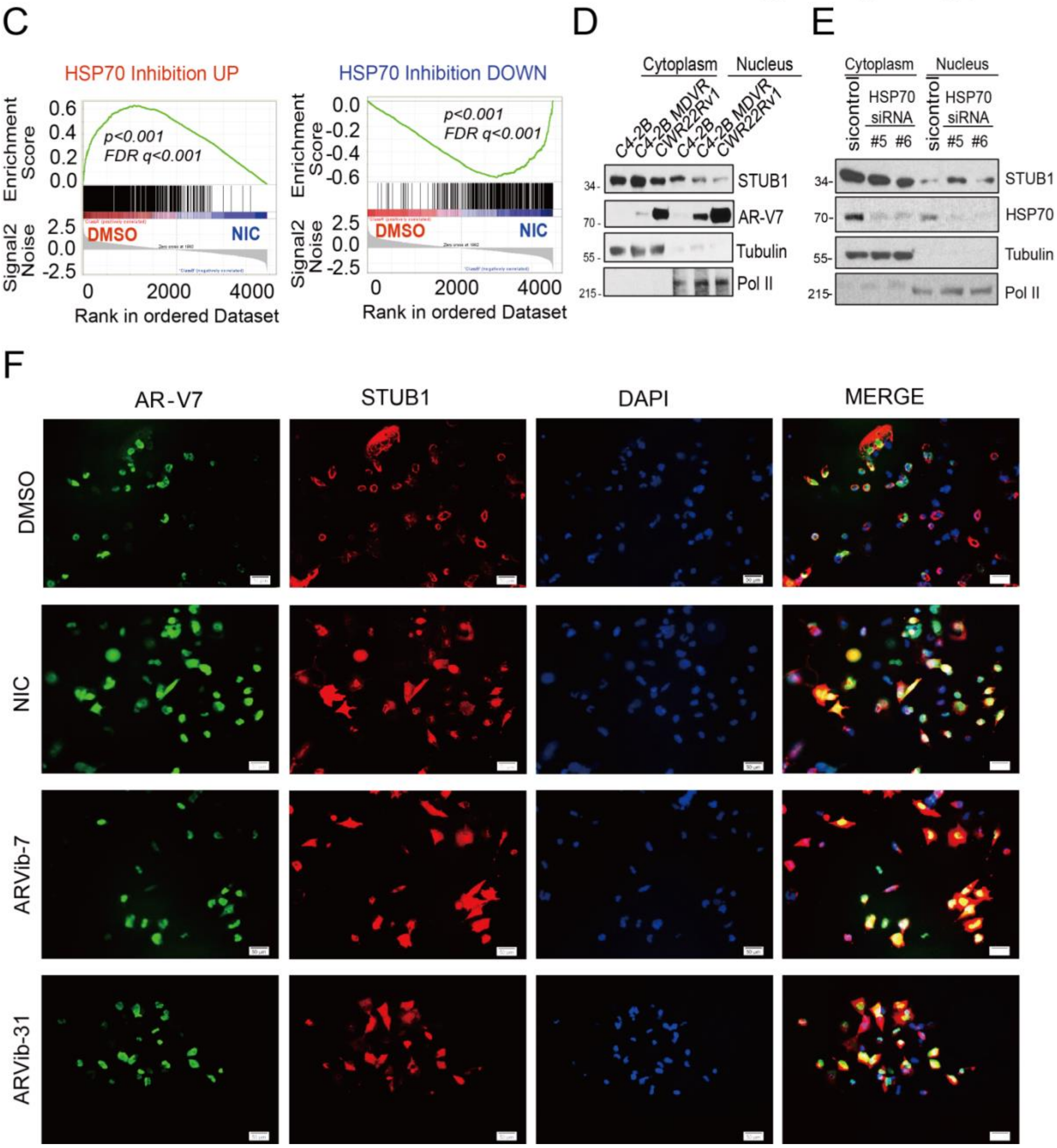

Supplementary Figure 4. ARVib modulates the HSP70/STUB1 complex. (A) HSP70 family member gene expression was suppressed by ARVib in C4-2B MDVR cells as demonstrated by RNA-seq data analysis. (**B**) C4-2B MDVR cells were treated with different doses of ARVib, total RNA was extracted and mRNA level of HSPA1B was examined by real-time PCR. * $p<0.05$. Results are the mean of three independent experiments (±S.D.). Statistical analysis was performed using two tailed Student's t test. **(C)** GSEA analysis reveals enrichment of the HSP70 inhibition signature in resistant cells treated with niclosamide. The signature was defined by genes with significant expression changes by HSP70 inhibition in prostate cancer cells. (**D**) Cytosolic and nuclear protein from C4-2B, C4-2B MDVR and CWR22Rv1 cells was extracted and subjected to western blot. (**E**) HSP70 was knocked down by two independent siRNA for 3 days in C4-2B MDVR cells, cytosolic and nuclear protein was extracted and subjected to western blot. (**F**) 293 cells were co-transfected with AR-V7, HSP70 and Flag-STUB1 for 3 days and then treated with DMSO or ARVib (1 μM) and 5 μM MG132 for 24 hours. AR-V7 and STUB1 were visualized by dual immunofluorescence staining.

Supplementary Figure 5

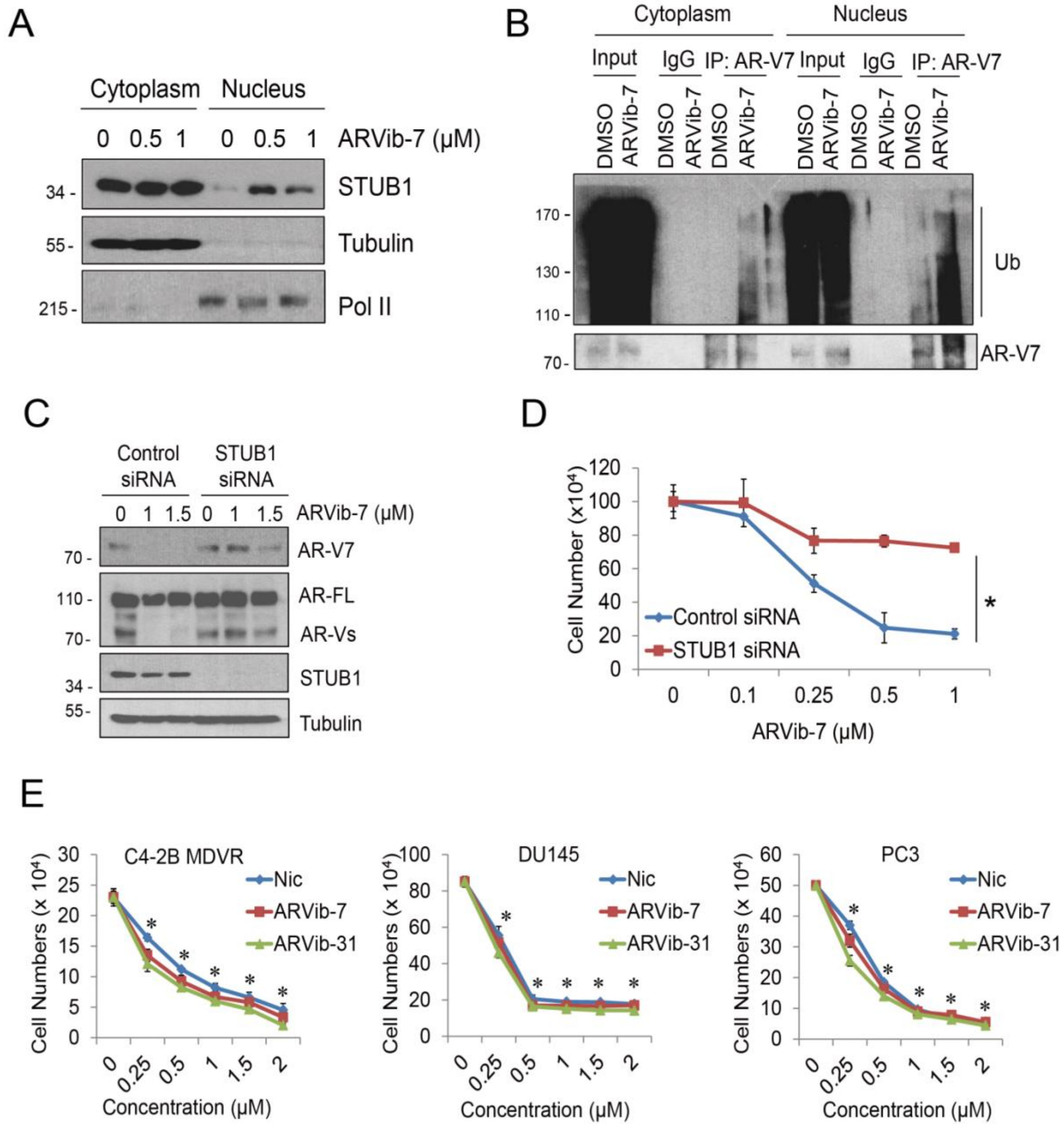

Supplementary Figure 5. ARVib degrades AR-V7 through STUB1. (A) C4-2B MDVR cells were treated with DMSO, 0.5 or 1.0 μM ARVib-7 16 hours and 5 μM MG132 for an additional 6 hours. Cytosolic and nuclear proteins were extracted and subjected to western blot. (**B**) C4-2B MDVR cells were treated with DMSO, 0.5 or 1.0 μM ARVib-7 16 hours and 5 μM MG132 for an additional 6 hours. Cytosolic and nuclear proteins were extracted and immunoprecipitated with AR-V7 antibody and blotted with anti-Ub and AR-V7 antibodies. (**C**) C4-2B MDVR cells were transiently transfected with STUB1 siRNA for 3 days and then

treated with different doses of ARVib-7 16 hours. Whole cell lysates were collected and subjected to western blot. (**D**) C4-2B MDVR cells were transiently transfected with STUB1 siRNA for 3 days and then treated with different doses of ARVib-7 for 3 days. Total cell numbers were determined. (**E**) C4-2B MDVR, DU145 and PC3 cells were treated with different dose of niclosamide, ARVib-7 or ARVib-31 for 3 days, total cell number were determined. * $p<0.05$. Results are the mean of three independent experiments (±S.D.).

Supplementary Figure 6

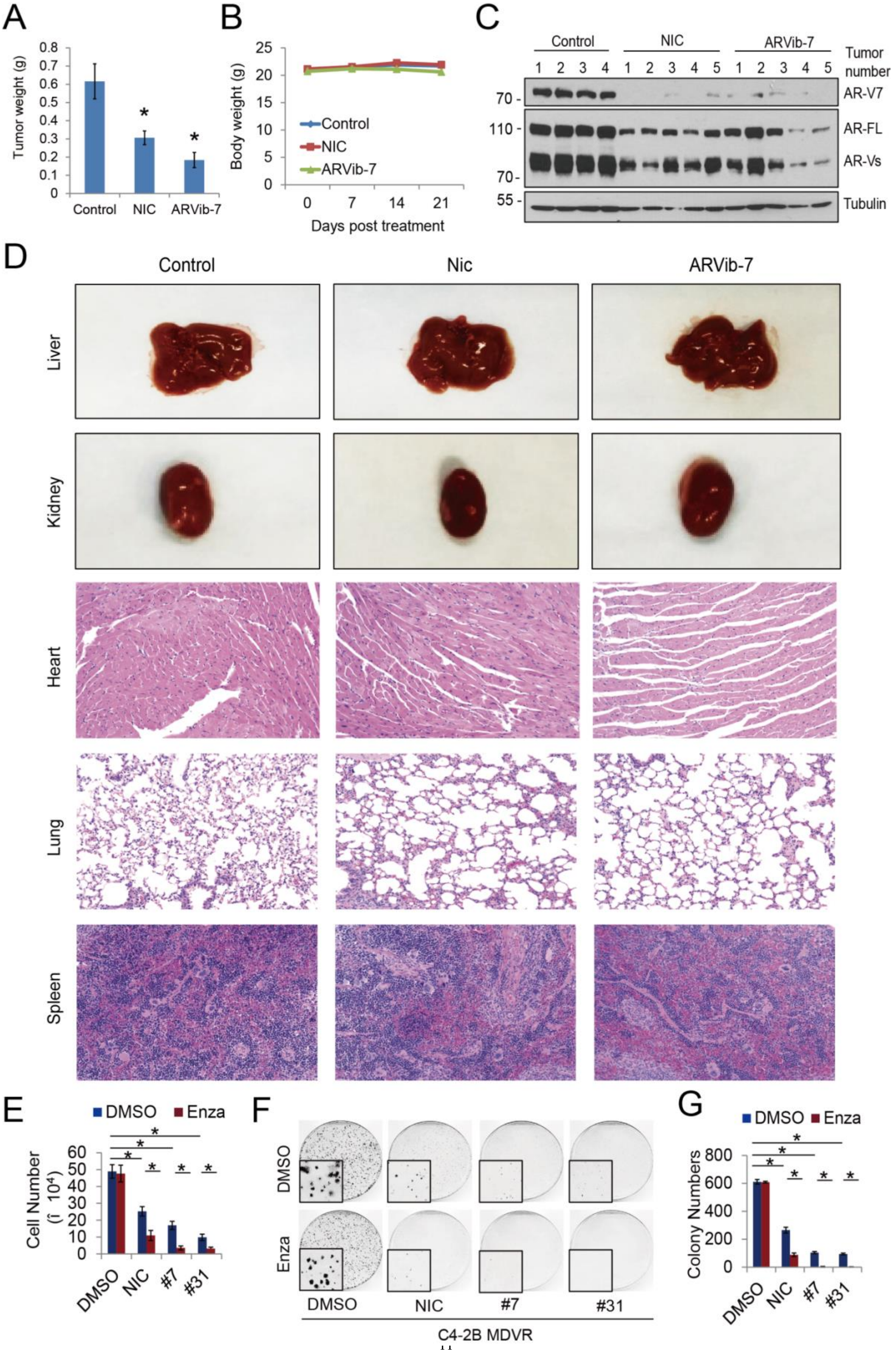

Supplementary Figure 6. ARVib suppresses prostate tumor growth and improves enzalutamide treatment (A) Mice bearing CWR22Rv1 xenografts were treated with vehicle control, niclosamide (25 mg/Kg i.p) or ARVib-7 (25 mg/Kg i.p) for 3 weeks (n=6). Tumors were collected and weighed. (B) Body weight was monitored twice per week. Data represent means ±S.D. from 7 tumors per group. (**C**) Tumor protein from each group was extracted and subjected to western blot. (**D**) Liver and kidney were harvested from LuCaP 35CR xenograft mice and photographed. H&E staining of heart, lung and spleen was performed. (**E**) C4-2B MDVR cells were treated with 0.25 μM niclosamide, ARVib-7 or ARVib-31 in combination with 20 μM enzalutamide for 3 days. Total cell numbers were determined. (**F-G**) C4-2B MDVR cells were treated with 0.25 μM niclosamide, ARVib-7 or ARVib-31 in combination with 20 μM enzalutamide. Clonogenic assay was performed and the number of colonies was determined. * $p<0.05$. Results are the mean of three independent experiments (±S.D.). Statistical analysis was performed using two tailed Student's t test.

Supplementary Table 1. Up regulated gene sets in ARVib treated C4-2B MDVR cells

NAME	NES	NOM p-value
HALLMARK_P53_PATHWAY	2.4256454	0.0000
HALLMARK_INFLAMMATORY_RESPONSE	2.3338137	0.0000
HALLMARK_HEME_METABOLISM	1.963814	0.0000
HALLMARK_IL6_JAK_STAT3_SIGNALING	1.9456544	0.0020
HALLMARK_UNFOLDED_PROTEIN_RESPONSE	1.9187012	0.0000
HALLMARK_INTERFERON_GAMMA_RESPONSE	1.9065014	0.0020
HALLMARK_CHOLESTEROL_HOMEOSTASIS	1.9041417	0.0040
HALLMARK_COAGULATION	1.8288968	0.0100
HALLMARK_HYPOXIA	1.8065017	0.0000
HALLMARK_APOPTOSIS	1.7748673	0.0000
KEGG_RIBOSOME	2.4177873	0.0000
KEGG_CYTOKINE_CYTOKINE_RECEPTOR_INTERACTION	2.0938303	0.0019
KEGG_SNARE_INTERACTIONS_IN_VESICULAR_TRANSPORT	1.9443306	0.0021
KEGG_LYSOSOME	1.7147795	0.0061
KEGG_NATURAL_KILLER_CELL_MEDIATED_CYTOTOXICITY	1.6509591	0.0262
KEGG_ERBB_SIGNALING_PATHWAY	1.5469512	0.0346
KEGG_CELL_ADHESION_MOLECULES_CAMS	1.5248582	0.0316
KEGG_LEUKOCYTE_TRANSENDOTHELIAL_MIGRATION	1.4979825	0.0481
REACTOME_PEPTIDE_CHAIN_ELONGATION	2.3380086	0.0000
REACTOME_SRP_DEPENDENT_COTRANSLATIONAL_PROTEIN_TARGETING_TO_MEMBRANE	2.1720405	0.0000
REACTOME_PHASE1_FUNCTIONALIZATION_OF_COMPOUNDS	2.0089476	0.0042
REACTOME_SIGNALING_BY_ERBB4	1.8289428	0.0019

REACTOME_PI3K_EVENTS_IN_ERBB2_SIGNALING	1.8224328	0.0157
REACTOME_UNFOLDED_PROTEIN_RESPONSE	1.8064979	0.0021
REACTOME_3_UTR_MEDIATED_TRANSLATIONAL_REGULATION	1.689577	0.0061
REACTOME_CYTOSOLIC_TRNA_AMINOACYLATION	1.6864245	0.0177
REACTOME_IMMUNOREGULATORY_INTERACTIONS_BETWEEN_A_LYMPHOID_AND_A_NON_LYMPHOID_CELL	1.6817012	0.0227
REACTOME_TRANS_GOLGI_NETWORK_VESICLE_BUDDING	1.6677707	0.0167
REACTOME_PI3K_EVENTS_IN_ERBB4_SIGNALING	1.6616333	0.0283
REACTOME_PERK_REGULATED_GENE_EXPRESSION	1.6543653	0.0236
REACTOME_INTERFERON_ALPHA_BETA_SIGNALING	1.6478027	0.0115
REACTOME_INTERFERON_GAMMA_SIGNALING	1.6074164	0.0300
REACTOME_TRANSLATION	1.6031276	0.0125
REACTOME_CLASS_B_2_SECRETIN_FAMILY_RECEPTORS	1.5839899	0.0211
REACTOME_DIABETES_PATHWAYS	1.5834861	0.0106
REACTOME_SIGNALING_BY_SCF_KIT	1.5730137	0.0479
REACTOME_GAB1_SIGNALOSOME	1.5647377	0.0439
REACTOME_ACTIVATION_OF_GENES_BY_ATF4	1.5551846	0.0494
REACTOME_TRANSPORT_TO_THE_GOLGI_AND_SUBSEQUENT_MODIFICATION	1.5487257	0.0391
REACTOME_GPCR_LIGAND_BINDING	1.5442389	0.0224
REACTOME_NONSENSE_MEDIATED_DECAY_ENHANCED_BY_THE_EXON_JUNCTION_COMPLEX	1.5106287	0.0217
REACTOME_SPHINGOLIPID_METABOLISM	1.4987527	0.0414
REACTOME_MEMBRANE_TRAFFICKING	1.4918388	0.0391
REACTOME_ADAPTIVE_IMMUNE_SYSTEM	1.3863758	0.0158
REACTOME_IMMUNE_SYSTEM	1.2900531	0.0498

Supplementary Table 2. Down regulated gene sets in ARVib treated C4-2B MDVR cells

NAME	NES	NOM p-value
HALLMARK_E2F_TARGETS	-3.6501343	0.0000
HALLMARK_G2M_CHECKPOINT	-3.453412	0.0000
HALLMARK_MYC_TARGETS_V2	-3.333848	0.0000
HALLMARK_MYC_TARGETS_V1	-3.0630283	0.0000
HALLMARK_ANDROGEN_RESPONSE	-2.3942077	0.0000
HALLMARK_MITOTIC_SPINDLE	-1.973954	0.0000
KEGG_DNA_REPLICATION	-2.8318264	0.0000
KEGG_CELL_CYCLE	-2.6476054	0.0000
KEGG_SPLICEOSOME	-2.4567487	0.0000
KEGG_SYSTEMIC_LUPUS_ERYTHEMATOSUS	-2.2802567	0.0000
KEGG_PURINE_METABOLISM	-2.103461	0.0000
KEGG_BASE_EXCISION_REPAIR	-2.075307	0.0000
KEGG_HOMOLOGOUS_RECOMBINATION	-1.9235047	0.0042
KEGG_PYRIMIDINE_METABOLISM	-1.8924199	0.0020
KEGG_PROGESTERONE_MEDIATED_OOCYTE_MATURATION	-1.7437338	0.0064
KEGG_DRUG_METABOLISM_CYTOCHROME_P450	-1.704726	0.0155
KEGG_NEUROACTIVE_LIGAND_RECEPTOR_INTERACTION	-1.6898865	0.0061
KEGG_NUCLEOTIDE_EXCISION_REPAIR	-1.6718376	0.0153
KEGG_OOCYTE_MEIOSIS	-1.6659161	0.0100

REACTOME_G1_S_TRANSITION	-3.429591	0.0000
REACTOME_CELL_CYCLE	-3.3122485	0.0000
REACTOME_DNA_REPLICATION	-3.3013022	0.0000
REACTOME_S_PHASE	-3.1981616	0.0000
REACTOME_CELL_CYCLE_MITOTIC	-3.1599967	0.0000
REACTOME_MITOTIC_G1_G1_S_PHASES	-3.1499	0.0000
REACTOME_MITOTIC_M_M_G1_PHASES	-3.1389034	0.0000
REACTOME_DNA_STRAND_ELONGATION	-3.0934153	0.0000
REACTOME_ACTIVATION_OF_THE_PRE_REPLICATIVE_COMPLEX	-3.0819383	0.0000
REACTOME_E2F_MEDIATED_REGULATION_OF_DNA_REPLICATION	-3.0707061	0.0000
REACTOME_M_G1_TRANSITION	-3.0217013	0.0000
REACTOME_G1_S_SPECIFIC_TRANSCRIPTION	-3.0041122	0.0000
REACTOME_G2_M_CHECKPOINTS	-2.9958324	0.0000
REACTOME_SYNTHESIS_OF_DNA	-2.9766903	0.0000
REACTOME_ACTIVATION_OF_ATR_IN_RESPONSE_TO_REPLICATION_STRESS	-2.975267	0.0000
REACTOME_CHROMOSOME_MAINTENANCE	-2.908514	0.0000
REACTOME_TELOMERE_MAINTENANCE	-2.894609	0.0000
REACTOME_MRNA_PROCESSING	-2.8396115	0.0000
REACTOME_PROCESSING_OF_CAPPED_INTRON_CONTAINING_PRE_MRNA	-2.807475	0.0000
REACTOME_EXTENSION_OF_TELOMERES	-2.7029057	0.0000
REACTOME_CELL_CYCLE_CHECKPOINTS	-2.6329043	0.0000
REACTOME_MEIOTIC_RECOMBINATION	-2.6324575	0.0000
REACTOME_G0_AND_EARLY_G1	-2.590677	0.0000
REACTOME_MRNA_SPLICING	-2.5731232	0.0000

REACTOME_TRANSCRIPTION	-2.5457945	0.0000
REACTOME_ASSEMBLY_OF_THE_PRE_REPLICATIVE_COMPLEX	-2.5291214	0.0000
REACTOME_MEIOSIS	-2.5270026	0.0000
REACTOME_DEPOSITION_OF_NEW_CENPA_CONTAINING_NUCLEOSOMES_AT_THE_CENTROMERE	-2.4929788	0.0000
REACTOME_LAGGING_STRAND_SYNTHESIS	-2.4711041	0.0000
REACTOME_RNA_POL_I_RNA_POL_III_AND_MITOCHONDRIAL_TRANSCRIPTION	-2.4568815	0.0000
REACTOME_MITOTIC_PROMETAPHASE	-2.4279478	0.0000
REACTOME_METABOLISM_OF_NON_CODING_RNA	-2.4173305	0.0000
REACTOME_RNA_POL_I_PROMOTER_OPENING	-2.3970208	0.0000
REACTOME_TRANSPORT_OF_MATURE_TRANSCRIPT_TO_CYTOPLASM	-2.3758795	0.0000
REACTOME_RNA_POL_I_TRANSCRIPTION	-2.357174	0.0000
REACTOME_ORC1_REMOVAL_FROM_CHROMATIN	-2.3454108	0.0000
REACTOME_PACKAGING_OF_TELOMERE_ENDS	-2.2868698	0.0021
REACTOME_DNA_REPAIR	-2.2341654	0.0000
REACTOME_AMYLOIDS	-2.225185	0.0000
REACTOME_MRNA_SPLICING_MINOR_PATHWAY	-2.1975927	0.0000
REACTOME_TRANSPORT_OF_MATURE_MRNA_DERIVED_FROM_AN_INTRONLESS_TRANSCRIPT	-2.1387322	0.0000
REACTOME_NEP_NS2_INTERACTS_WITH_THE_CELLULAR_EXPORT_MACHINERY	-2.1308575	0.0000
REACTOME_CLEAVAGE_OF_GROWING_TRANSCRIPT_IN_THE_TERMINATION_REGION_	-2.1239073	0.0000
REACTOME_RNA_POL_II_TRANSCRIPTION	-2.11891	0.0000
REACTOME_FORMATION_OF_TUBULIN_FOLDING_INTERMEDIATES_BY_CCT_TRIC	-2.1029038	0.0020
REACTOME_MEIOTIC_SYNAPSIS	-2.0990524	0.0000
REACTOME_TRANSPORT_OF_RIBONUCLEOPROTEINS_INTO_THE_HOST_NUCLEUS	-2.0971913	0.0000

REACTOME_GLUCOSE_TRANSPORT	-2.0636134	0.0000
REACTOME_PREFOLDIN_MEDIATED_TRANSFER_OF_SUBSTRATE_TO_CCT_TRIC	-2.0607743	0.0019
REACTOME_METABOLISM_OF_NUCLEOTIDES	-2.0285134	0.0000
REACTOME_REGULATION_OF_MITOTIC_CELL_CYCLE	-2.027396	0.0020
REACTOME_REGULATION_OF_GLUCOKINASE_BY_GLUCOKINASE_REGULATORY_PROTEIN	-2.0235674	0.0000
REACTOME_MITOTIC_G2_G2_M_PHASES	-1.9888797	0.0020
REACTOME_HIV_LIFE_CYCLE	-1.9668924	0.0000
REACTOME_CYCLIN_E_ASSOCIATED_EVENTS_DURING_G1_S_TRANSITION_	-1.9609337	0.0000
REACTOME_PURINE_METABOLISM	-1.9537992	0.0021
REACTOME_INTERACTIONS_OF_VPR_WITH_HOST_CELLULAR_PROTEINS	-1.9461582	0.0083
REACTOME_DOUBLE_STRAND_BREAK_REPAIR	-1.9189279	0.0019
REACTOME_MRNA_3_END_PROCESSING	-1.905243	0.0041
REACTOME_LATE_PHASE_OF_HIV_LIFE_CYCLE	-1.8351958	0.0000
REACTOME_GLOBAL_GENOMIC_NER_GG_NER	-1.7530818	0.0181
REACTOME_TRANSCRIPTION_COUPLED_NER_TC_NER	-1.747458	0.0157
REACTOME_GLYCOLYSIS	-1.7444582	0.0115
REACTOME_RNA_POL_II_TRANSCRIPTION_PRE_INITIATION_AND_PROMOTER_OPENING	-1.7242773	0.0142
REACTOME_PROCESSING_OF_CAPPED_INTRONLESS_PRE_MRNA	-1.7199014	0.0080
REACTOME_CDT1_ASSOCIATION_WITH_THE_CDC6_ORC_ORIGIN_COMPLEX	-1.6763219	0.0283
REACTOME_RNA_POL_III_TRANSCRIPTION	-1.6581949	0.0060
REACTOME_FACTORS_INVOLVED_IN_MEGAKARYOCYTE_DEVELOPMENT_AND_PLATELET_PRODUCTION	-1.5879005	0.0242
REACTOME_HIV_INFECTION	-1.5503463	0.0137
REACTOME_ANTIVIRAL_MECHANISM_BY_IFN_STIMULATED_GENES	-1.5242358	0.0297

REACTOME_METABOLISM_OF_RNA	-1.4837075	0.0121
REACTOME_RECRUITMENT_OF_MITOTIC_CENTROSOME_PROTEINS_AND_COMPLEXES	-1.4833018	0.0363
REACTOME_HOST_INTERACTIONS_OF_HIV_FACTORS	-1.4764512	0.0394

Primer list for the qRT-PCR

	Forward	Reverse
AR-V7	AACAGAAGTACCTGTGCGCC	TCAGGGTCTGGTCATTTTGA
AR-FL	AAGCCAGAGCTGTGCAGATGA	TGTCCTGCAGCCACTGGTTC
HSP70 (HSPA1B)	TGGACTGTTGGGACTCAAGGAC	GGAACGAAACACCCTTACAGTATCA
KLK2	CAACATCTGGAGGGGAAAGGG	AGGCCAAGTGATGCCAGAAC
KLK3	GCCCTGCCCGAAAGG	GATCCACTTCCGGTAATGCA
FKBP5	GGGAAGATAGTGTCCTGGTTAG	GCAGTCTTGCAGCCTTATTC
NKX3-1	CCGAGACGCTGGCAGAGACC	GCTTAGGGGTTTGGGGAAG
UBE2C	TGGTCTGCCCTGTATGATGT	AAAAGCTGTGGG GTTTTTCC
Myc	TGAGGAGACACCGCCCAC	CAACATCGATTTCTTCCTCATC
ACTIN	AGAACTGGCCCTTCTTGGAGG	GTTTTTATGTTCCTCTATGGG
